# Leader Communication Techniques: Analyzing the Effects on Followers’ Cognitions, Affect, and Behavior

**DOI:** 10.3390/bs15081018

**Published:** 2025-07-27

**Authors:** Chantal Utzinger, Anna Luca Heimann, Fabiola H. Gerpott, Hubert Annen, Martin Kleinmann

**Affiliations:** 1 Department of Military Psychology and Pedagogics, Military Academy at ETH Zurich, 8903 Birmensdorf, Switzerland; 2 Department of Psychology, University of Zurich, 8050 Zürich, Switzerland; 3 Management Group, WHU Otto Beisheim School of Management, 40233 Düsseldorf, Germany

**Keywords:** leader communication, follower outcomes, vignette experiment

## Abstract

How leaders communicate with followers is a core element of leadership. However, research on leader communication techniques remains fragmented, limiting our understanding of their differential effects on follower cognition, affect, and behavior. To facilitate systematic research comparing the effects of different communication techniques, we propose a framework for leader communication techniques. We hypothesize that different communication techniques can be categorized into cognitive, affective, and behavioral leader communication techniques that influence cognitive, affective, and behavioral follower outcomes, respectively. To test this assumption, we developed and pre-tested video vignette material, which we then used in a between-subjects experiment in the military context. We assigned 368 military recruits to one of the three conditions (cognitive versus affective versus behavioral) to examine how these techniques relate to proximal and distal follower outcomes. We found few differences in the impact of cognitive, affective, and behavioral leader communication techniques on follower outcomes. The leader was perceived similarly, regardless of the communication techniques used. Only for specific affective follower outcomes (i.e., warmth and charisma of the leader), affective leader communication techniques were more effective than cognitive and behavioral techniques. We discuss implications for leader communication research, outline practical implications for leaders, and propose directions for future research.

## 1. Introduction

At its core, leadership refers to the process of influencing others’ cognition, affect, and behaviors (Avolio et al., 2009; Rockstuhl et al., 2012; Yukl, 2012). Leaders primarily exert influence through communication (Antonakis et al., 2016; E. H. Liu et al., 2023), defined as the process of using signs and language to understand each other (Fairhurst & Connaughton, 2014). In fact, leaders spend a significant amount of their working time communicating with their followers (E. H. Liu et al., 2023; Tengblad, 2006). In line with this, research has shown that specific leader communication techniques are associated with relevant outcomes such as followers’ perceptions of their leader and followers’ behavior on the job (Antonakis et al., 2022; Gkalitsiou & Kotsopoulos, 2023; Meyer et al., 2016; Weiss et al., 2018). Imagine a leader briefing their team before a project. The leader might ask questions to check comprehension and invite input, praise followers to acknowledge contributions and strengthen motivation, and set goals to align the followers on the desired outcome. These specific communication behaviors can shape how followers process the task cognitively (Do I understand what I need to do?), respond emotionally (Do I feel motivated?), and act behaviorally (Am I ready to execute the task?).

While research on communication techniques—defined as specific methods, meanings, signs, and language used to convey information (Addimando, 2024)—has highlighted the important role of communication in leadership, two issues characterize this nascent research stream. First, this field so far remains largely fragmented, with different studies focusing on different communication techniques (e.g., Meyer et al., 2016; Weiss et al., 2018; Hemshorn de Sanchez et al., 2024). This fragmentation is problematic because it impedes cumulative knowledge building: without a comprehensive framework that integrates various communication techniques, findings remain difficult to compare, leaving scholars and practitioners without clear guidance on which techniques are most effective to influence followers’ cognition, affect, and behaviors. For example, one study might focus on how *asking questions* influences follower understanding, while another examines the impact of *praising* on follower motivation—without a shared framework to meaningfully relate these findings. Second, it is outweighed by the overwhelming volume of studies focusing on broad leadership styles rather than specific communication techniques (Fischer & Sitkin, 2023; Meyer et al., 2016). In other words, despite the valuable preliminary insights provided, the exploration of communication techniques has not yet received the same level of attention or depth of analysis as the more extensively studied domain of leadership styles (Fischer & Sitkin, 2023; Meyer et al., 2016). As a result, we lack an understanding of which concrete communication behaviors matter for which follower outcomes. This oversight stands in contrast with recent calls within the field for a stronger focus on leadership behavior (Banks et al., 2023; Fischer & Sitkin, 2023), that is, “it is necessary to study actual behavior” (Baumeister et al., 2007, p. 400).

The present study proposes an overarching model of leader communication techniques, with the purpose of first categorizing and, second, empirically testing the effectiveness of different communication techniques in the context of leadership. We define leader communication techniques as verbal, paraverbal, and nonverbal behavior at the micro level that a leader can use to influence how followers think, feel, and behave. While verbal communication techniques involve the use of language (e.g., questions or metaphors), paraverbal communication techniques refer to tone, pitch, and speed, and nonverbal communication techniques include facial expressions and gestures (Sundaram & Webster, 2000). Leaders may use these communication techniques with the intent to influence followers’ cognitive reactions (e.g., their task understanding), affective reactions (e.g., their task motivation), or behavioral reactions (e.g., their behavioral intention to complete a task; Avolio et al., 2009). Accordingly, we organize leader communication techniques that have been studied in previous research (e.g., Van Quaquebeke & Felps, 2018; Weiss et al., 2018; Hemshorn de Sanchez et al., 2022, 2024) into a framework that distinguishes between communication channels on the one hand (i.e., verbal, paraverbal, or nonverbal communication) and intended follower impact on the other hand (i.e., communication that seeks to influence followers’ cognition, affect, or behavior). Specifically, we hypothesize that cognitive leader communication techniques (e.g., asking questions) are particularly effective in shaping followers’ cognitive responses (e.g., task understanding), affective leader communication techniques (e.g., praising others) in influencing affective responses (e.g., task motivation), and behavioral leader communication techniques (e.g., giving precise instructions) in eliciting behavioral follower outcomes (e.g., task performance).

We contribute to the leadership literature in three ways. First, we extend knowledge about leader communication techniques. By synthesizing the fragmented research on leadership communication techniques into an overarching framework, we can compare the effects of specific communication techniques and, as a result, understand which leadership communication techniques are relevant for addressing different follower outcomes. We not only develop such a framework but also validate it through a survey with subject matter experts, followed by experimental studies to empirically test the effect of different categories of leader communication techniques on follower outcomes.

Second, by focusing on specific communication techniques at the micro level, we respond to calls for more research on specific leader behaviors rather than broad leadership styles and categories of behavior (Banks et al., 2023; Fischer & Sitkin, 2023). While it is well established in leadership research that communication plays a central role, communication is often treated as just one component within broader leadership styles (E. H. Liu et al., 2023). As a result, the question of which specific communication behaviors enable leaders to be effective in their interactions with followers remains unanswered.

Third, we also offer a practical contribution as there are many different types of communication training programs (Antonakis et al., 2011; Avolio et al., 2009). To develop leaders in communication techniques, providers need to know which communication techniques are effective for leaders for influencing different follower outcomes.

## 2. Leader Communication

Earlier leadership research has suggested constructs and taxonomies describing broad categories of leadership behavior to understand what makes a leader effective in the interaction with their followers (de Vries et al., 2010; DeRue et al., 2011; Dumdum et al., 2013). Most of these behavioral constructs and taxonomies acknowledge that leader communication is of importance for effective leadership (Antonakis et al., 2016; Bass, 1985; Yukl et al., 2002). For example, according to transformational leadership theory, leaders provide intellectual stimulation, express high performance expectations, and provide individual support (Bass, 1985; Podsakoff et al., 1990). As a leader, a predominant way to implement these aspects of transformational leadership is by communicating with followers (Lehmann-Willenbrock et al., 2015). As another illustration, charismatic leadership theory emphasizes the articulation of a leader’s vision and values through symbolic language and emotional expressions (Antonakis et al., 2016). Moreover, communication is part of Yukl’s (2012) hierarchical taxonomy of leadership behavior (Yukl, 2012; Yukl et al., 2002). For instance, the task-oriented leadership category includes behaviors such as explaining tasks and responsibilities (Yukl, 2012; Yukl et al., 2002). In the relations-oriented leadership category, they include behaviors such as expressing encouragement, and the change-oriented leadership category encompasses behaviors such as communicating a clear vision.

While research on leadership styles has advanced our understanding of effective leadership (Dumdum et al., 2013; Judge & Piccolo, 2004), these constructs have been criticized because they are broad and do not allow for identifying single behaviors that help leaders to be effective when interacting with their followers (Banks et al., 2023; van Knippenberg & Sitkin, 2013). Scholars are therefore calling for more research on specific behaviors, meaning leader behavior at the micro-level (Hemshorn de Sanchez et al., 2022; Banks et al., 2023), which is also referred to as the basic building blocks of leadership (Fischer et al., 2023; Van Quaquebeke & Felps, 2018). This is important because “a focus on discrete, identifiable behaviors allows for meaningful scholarly conversations about what leaders actually do” (Fischer et al., 2023, p. 2).

Leader communication techniques can be considered leader communication behavior at the micro-level because they comprise specific observable verbal, non-verbal, and paraverbal conduct of leaders in interaction with their followers and may influence how followers think, feel, and act (E. H. Liu et al., 2023; Meyer et al., 2016). Only recently, scholars have started to offer a clear definition of what leader communication is “textual, verbal, and embodied signals that leaders deliver to others, both purposefully and unintentionally, with the power to reveal aspects of leaders themselves, predict leadership outcomes, and affect others” (E. H. Liu et al., 2023, p. 2). We approach the framework development based on this definition and thus take on a leader-centric perspective on leader communication (Fairhurst & Connaughton, 2014).

### 2.1. Outcomes of Leader Communication

There are multiple taxonomies for categorizing relevant outcomes in the leadership context (Avolio et al., 2009; DeRue et al., 2011; Hiller et al., 2011). In the field of leadership interventions, outcomes are commonly grouped into three broad categories: cognition (i.e., followers’ thoughts and perceptions), affect (i.e., followers’ emotions and relational experiences), and behavior (i.e., followers’ actions and performance; Avolio et al., 2009). We adopt this categorization not only because it is widely used in leadership intervention research but also because we see leader communication techniques as micro-level interventions—deliberate communicative acts through which leaders attempt to shape followers’ experiences and responses (see also E. H. Liu et al., 2023). From this perspective, leader communication techniques do not merely transmit information but serve as intentional efforts to influence follower outcomes, thereby aligning with the intervention logic outlined by Avolio et al. (2009). As such, the cognitive–affective–behavioral categorization provides a parsimonious yet comprehensive structure for integrating relevant outcomes. Cognitive follower outcomes refer to followers’ mental perceptions, understanding, and thoughts; this includes how followers perceive information, process it, and make decisions (Avolio et al., 2009). Research on leader communication frequently examines the mental perceptions of followers (Antonakis et al., 2011; Brandmo et al., 2021). For example, a study on leader communication training found that followers perceive their leader as more competent after the training (Antonakis et al., 2011), which reflects a cognitive follower outcome. Another study found that when leaders used frequent feedback, followers perceived their jobs as more clearly defined (Brandmo et al., 2021), which can also be understood as a cognitive follower outcome.

Affective follower outcomes in the workplace refer to followers’ emotions toward their leader, work colleagues, tasks, or organization (Avolio et al., 2009; Pearsall et al., 2009); this includes how followers feel about the leader, the task, and themselves. For example, a study on using metaphors for communication has shown that they create vivid images and elicit emotional reactions (Mio, 1997; Raju et al., 2025). Such emotional reactions can include followers liking their leader (Antonakis et al., 2011) or followers feeling that the leader is more charismatic (Grabo & van Vugt, 2016; Maran et al., 2019). For example, when leaders have a lot of eye contact with their followers, followers tend to feel that their leader is charismatic (Maran et al., 2019), which can be understood as an affective outcome. Another study on leader communication found that the manner in which a leader communicates has a positive impact on followers’ sense of belonging (Y. Liu et al., 2022), which we also understand as an affective follower outcome.

Lastly, behavioral follower outcomes refer to followers’ actions that are observable and contribute to the functioning of teams, projects, or organizations (Avolio et al., 2009; Harari et al., 2016); this includes followers’ performance-relevant behavior and behavioral intentions. Several studies examined whether followers’ performance improves depending on how the leader communicates. The findings indicated that when leaders use communication techniques such as setting goals, followers improve their performance (Antonakis et al., 2022; Meslec et al., 2020). Others have also investigated whether, and for how long, car drivers adhere to the instructions provided by a navigation system—a form of leadership guiding drivers’ actions and decisions (Niebuhr & Michalsky, 2019). The study showed that when the leading navigation system had a more appealing voice, drivers followed navigation instructions for a longer period (even if they were incorrect; Niebuhr & Michalsky, 2019), illustrating a form of behavioral follower outcome.

Leadership is a process that entails some outcomes occurring relatively immediately (i.e., proximal outcomes), while others happen less immediately (i.e., distal outcomes). To capture this, we categorize the cognitive, affective, and behavioral follower outcomes as either proximal or distal outcomes (see also Day & Dragoni, 2015; Heavey et al., 2013). We consider proximal outcomes in followers to be those outcomes of communication techniques that occur immediately after the follower’s contact with the leader; that is, we expect them to occur in direct response to a one-time contact with the leader. Distal outcomes, on the other hand, capture outcomes that occur when participants interact with their leaders across subsequent, distinct scenarios, often requiring situational diversity to observe an effect. For example, followers’ task understanding or task motivation can be considered proximal outcomes of leader communication, as they are reactions to the leader communication techniques in one situation. In contrast, occupational self-efficacy and affective commitment can be considered distal follower outcomes, which reflect deeper, more enduring effects of the leader’s influence on followers.

### 2.2. A Framework for Leader Communication Techniques

Scholars have demonstrated the positive effects of specific leader communication techniques such as asking questions or using personal pronouns (e.g., Meyer et al., 2016; Mio et al., 2005; Van Quaquebeke & Felps, 2018; Weiss et al., 2018). However, there is no overarching framework that categorizes these techniques and compares their effect on different outcomes (i.e., follower cognition, affect, and behavior). While existing research in communication has established a classification based on the *form* of communication—namely verbal, paraverbal, and nonverbal channels (e.g., Sundaram & Webster, 2000)—there is currently no widely accepted classification based on the *intended impact* or content of the communication techniques. To address this, we build our framework on two well-established theoretical foundations. First, the cognitive–affective–behavioral taxonomy draws from extensive leadership intervention research identifying these three dimensions as core domains of follower outcomes—reflecting how followers think (cognition), feel (affect), and act (behavior) in response to leadership (Avolio et al., 2009; Kim et al., 2020). This taxonomy offers a parsimonious yet comprehensive lens through which to examine the effects of leader communication techniques. Second, communication research includes channels of communication—namely verbal, para-verbal, and nonverbal modes (Sundaram & Webster, 2000; Burgoon et al., 2021). These channels reflect distinct forms of information delivery: linguistic elements (verbal), aspects of the voice (paraverbal), and forms of movement of the body and face (nonverbal). Building on these two theoretical foundations, we distinguish leader communication techniques according to their impact level—that is, the type of follower outcome they are meant to influence: cognitive (e.g., thoughts, perceptions), affective (e.g., emotions, relational experiences), or behavioral (e.g., actions, performance)—as well as the communication channel used (verbal, para-verbal, or nonverbal). While a single technique may theoretically affect multiple types of outcomes, our allocation of the techniques focuses on the category in which its strongest or most direct impact is expected.

This dual lens enables us to analyze leader communication both in terms of *what* it aims to change in followers and *how* it is conveyed. Accordingly, the present study introduces an overarching framework of leader communication techniques (see Table 1) that considers (a) the communication channel (verbal, paraverbal, or nonverbal communication) and (b) the intended follower impact (cognitive, affective, or behavioral impact). We focus primarily on communication techniques that are expected to have a positive effect on followers because our objective is to gain insight into the ways in which leaders can promote followers’ cognitive, affective, and behavioral reactions within the workplace. While we recognize that communication is a two-way process and not solely directed from leader to follower (see Fairhurst & Connaughton, 2014 for a more detailed discussion), research on leader–follower interactions suggests a simultaneous influence process, rendering the sequence of perspectives less relevant (Güntner et al., 2020). Therefore, a unidirectional perspective serves as a practical starting point for our study, enabling us to develop and test the framework effectively. Consequently, communication behaviors that are direct reactions to follower behaviors (e.g., listening, Kluger et al., 2024; attentiveness, Decuypere & Pircher Verdorfer, 2022) are not included in the framework.

#### 2.2.1. Communication Channel

Leaders can communicate through multiple channels: verbal, paraverbal, and non-verbal (Sundaram & Webster, 2000). Verbal leader communication techniques refer to linguistic elements, such as asking questions, talking with images, or using inclusive forms of language (Mio et al., 2005; Van Quaquebeke & Felps, 2018; Weiss et al., 2018). For example, by asking questions in a respectful way, leaders can invite followers to share their thoughts (Van Quaquebeke & Felps, 2018), or they can promote information exchange (Meyer et al., 2016).

Paraverbal leader communication techniques refer to aspects of the voice, for example, voice pitch, speech tempo, and pauses in speech (Sundaram & Webster, 2000). Research on vocal characteristics has revealed that followers tend to prefer and elect male and female leaders with a lower voice pitch over those with a higher voice pitch (Anderson & Klofstad, 2012; Klofstad, 2016).

Nonverbal leader communication techniques comprise actions that accompany verbal communication, such as facial expressions, eye contact, gestures, or body postures (Sundaram & Webster, 2000). For example, a study showed that those leaders who maintain eye contact with their followers for more extended periods and more frequently are perceived as both more charismatic and more prototypical of their position by their followers (Maran et al., 2019).

#### 2.2.2. Follower Impact

In line with prior research (Avolio et al., 2009; Kim et al., 2020), we differentiate between cognitive, affective, and behavioral follower outcomes, and we argue that leader communication techniques can be purposefully used to target these outcomes (e.g., E. H. Liu et al., 2023). Therefore, this research proposes a categorization of leader communication techniques based on their primary intended target: followers’ cognition, affect, or behavior. This categorization forms the basis of our empirical research.

Cognitive leader communication techniques are conceptualized as methods, signs, or language that a leader uses to influence followers’ mental perceptions, thoughts, and understanding of tasks and people (see also Berson et al., 2015; Hodgkinson & Healey, 2008; Meyer et al., 2016). For example, leader communication techniques such as *explaining*, *asking questions*, and *expressing moral conviction* are assumed to be cognitive in nature because they provide task-relevant information, prompt reflection, and guide interpretation (Antonakis et al., 2016; Van Quaquebeke & Felps, 2018). These cognitive techniques are not assumed to produce cognitive outcomes by definition; rather, they are hypothesized to do so based on the assumption—grounded in leadership psychology and leader communication—that leaders’ behaviors shape followers’ cognitive reactions (e.g., Avolio et al., 2009; E. H. Liu et al., 2023). As leadership is, to some extent, anchored in the minds of organizational members (Lord & Emrich, 2000), and leaders are thought to shape followers’ interpretations of their environment, we posit that leader communication techniques that structure or direct attention on the task at hand or the specific content of an issue are likely to influence cognitive outcomes. Accordingly, we propose the following hypothesis:

**Hypothesis** **1.** *Cognitive leader communication techniques will influence proximal cognitive follower outcomes (i.e., followers’ task understanding, followers’ perception of task relevance, followers’ lack of role ambiguity, leader clarity, and leader competence) and distal cognitive follower outcomes (i.e., followers’ occupational self-efficacy, followers’ attitudes toward job tasks, leader endorsement, and leader persuasiveness).*

Affective leader communication techniques are conceptualized as methods, signs, or language that a leader uses to influence followers’ emotions and strengthen the leader–follower relationship (see also Antonakis et al., 2011; Emrich et al., 2001; Maran et al., 2019). For example, leader communication techniques such as *praising others, agreeing*, and keeping *eye contact* are assumed to be affective in nature because they provide supportiveness, approval, and relational closeness (Emrich et al., 2001; Maran et al., 2019). These affective techniques are not assumed to produce affective outcomes by definition; rather, they are hypothesized to do so based on the assumption that leadership is an emotional process, in which leaders’ behaviors elicit emotional reactions in their followers (Dasborough & Ashkanasy, 2002). Because affective techniques emphasize the relationship between leader and follower and convey emotional cues, they are expected to shape followers’ motivational states and relations. Accordingly, we propose the following hypothesis:

**Hypothesis** **2.** *Affective leader communication techniques will influence proximal affective follower outcomes (i.e., followers’ task motivation, followers’ sense of belonging, and leader warmth) and distal affective follower outcomes (i.e., followers’ self-esteem, followers’ affective organizational commitment, leader liking, and leader charisma).*

Behavioral leader communication techniques are conceptualized as methods, signs, or language that a leader uses to influence followers’ behavior directly (see also Antonakis et al., 2022; Latham & Locke, 2007; Locke et al., 1981). For example, leader communication techniques such as *setting goals*, *making a proposition*, and *giving precise instructions* are assumed to be behavioral in nature because they provide direction, clarify expectations, and prompt action (Antonakis et al., 2022; Latham & Locke, 2007). These behavioral techniques are not assumed to produce behavioral outcomes by definition; rather, they are hypothesized to do so based on the assumption that an essential function of leadership is to guide and influence followers’ behaviors toward desired objectives (Yukl, 2012; Yukl et al., 2002). Because behavioral techniques emphasize task execution and action-taking, they are expected to shape followers’ behavioral intentions and actual behavior. Accordingly, we propose the following hypothesis:

**Hypothesis** **3.** *Behavioral leader communication techniques will influence proximal behavioral follower outcomes (i.e., followers’ behavioral intention to perform a task) and distal behavioral follower outcomes (i.e., followers’ task performance, followers’ intention to change behavior, and leader following).*

## 3. Methods

The objective of the present research was to develop and test a framework of leader communication techniques. To this end, we conducted a survey with subject matter experts, followed by two pre-studies and a main study.

More specifically, we first organized the communication techniques that have been studied in previous research into a framework that distinguishes between communication channels on the one hand (i.e., verbal, paraverbal, or nonverbal communication) and follower impact on the other hand (i.e., communication that influences followers’ cognition, affect, or behavior). We then administered a survey to subject matter experts for the purpose of categorizing communication techniques according to their impact on followers. Description and results of the subject matter experts survey can be found online (https://researchbox.org/4357, accessed on 18 June 2025). Subsequently, we ran two pre-studies to develop and test the study materials for the main study. In the main study, we conducted an experimental vignette study to test whether the cognitive, affective, or behavioral leader communication techniques impact followers’ cognition, affect, or behavior.

We conducted the two pre-studies and the main study in a military setting (i.e., Swiss Armed Forces), similar to previous leadership studies (e.g., LePine et al., 2016; Sefidan et al., 2021). In Switzerland, all male Swiss citizens must serve in the military (Federal authorities of the Swiss confederation, 2024). We chose this setting because, especially in a context where it cannot be assumed that all followers (i.e., military recruits) are motivated, leaders need to communicate effectively and convincingly.

Prior to participation in the studies, we provided the participants with comprehensive information. That is, (1) we informed participants that participation was voluntary, (2) we assured participants that all surveys are anonymous, and (3) we clarified that the study results would not contain any individual- or group-specific information. Participants then gave their informed consent to participate in the study in written form by checking a consent box in the online surveys that were used for data collection (Newman et al., 2021).

### 3.1. Pre-Studies 1 and 2: Vignette Development

To test the proposed framework, we designed a vignette experiment. In vignette experiments, participants are presented with fictitious scenarios (Atzmüller & Steiner, 2010). Subsequently, participants typically respond to questions assessing their reactions to this scenario (Aguinis & Bradley, 2014). The vignette method enables us to manipulate the independent variable (e.g., cognitive, affective, and behavioral leader communication techniques) while keeping all other factors constant (e.g., all other attributes and behaviors of the leader), allowing for the study of causal effects on dependent variables (e.g., follower outcomes; Aguinis & Bradley, 2014).

We designed vignettes for three conditions, namely (1) cognitive, (2) affective, and (3) behavioral leader communication techniques. For each condition, we created three vignettes describing different scenarios. This resulted in the development of nine vignettes (three conditions × three scenarios).

#### 3.1.1. Scenarios

We created vignettes for three different scenarios that are common in the military context in which the present study was conducted. In each of these three scenarios, a military leader with the rank of a lieutenant was talking to the recruits in his platoon and persuading them about the importance of different topics, namely (1) room tidiness, (2) marching drills, and (3) voluntary continuation after basic military training. We chose these three scenarios because they were frequently mentioned in conversations with military experts, they are common topics in military service and training, and every recruit is regularly confronted with them. The leader was the same fictitious lieutenant in each scenario.

In the scenario about the importance of *room tidiness*, the lieutenant explains the importance of maintaining a tidy room in the military context. He attempts to persuade the recruits to adhere to this standard in the future.

In the scenario of *voluntary marching drills*, the lieutenant illustrates the significance of practicing marching drills and the recruits’ active engagement. Marching drills constitute a fundamental component of basic military training. During these exercises, recruits are instructed in various types of marching, formations, and routes. The lieutenant provides the orders, and the recruits execute them in unison.

In the scenario of *voluntary continuation after basic military training*, the lieutenant illustrates the advantages and possibilities of the military cadre training. After the mandatory basic military training, Swiss soldiers must continue to serve the armed forces for three weeks each year (Federal authorities of the Swiss confederation, 2025). Going beyond this, they can also voluntarily pursue a military career, which allows them to be promoted and take on leadership roles (Swiss Armed Forces, 2022). The Swiss Armed Forces rely on soldiers taking leadership roles, and therefore, they try to convince recruits to decide on a leadership role already during basic training (Federal authorities of the Swiss confederation, 2025). However, it is challenging to identify a sufficient number of individuals who are willing to serve as leaders voluntarily. Therefore, in the scenario *voluntary continuation after basic military training*, the lieutenant seeks to persuade the recruits to continue their service as a leader and demonstrate the advantages of doing so.

#### 3.1.2. Vignette Structure

Each individual vignette encompassed only the leader communication techniques of one condition (i.e., either cognitive, affective, or behavioral techniques). To achieve this objective, we included the five most effective leader communication techniques from each category into each vignette of the respective condition. We used the mean effectiveness scores from the subject matter experts’ ratings from the survey to identify the five most effective leader communication techniques for each condition. The most effective leader communication techniques for each condition are printed in bold in Table 1. If a leader communication technique scored among the five most effective techniques for more than one category (cognitive, affective, and behavioral impact level), we assigned the technique only to the follower impact level for which the communication technique had the highest effectiveness rating. In cases in which two leader communication techniques had the same effectiveness rating, we decided on the technique with the lower standard deviation. We list the means and standard deviations for all effectiveness scores in Table S1 in the Supplementary Materials.

Each vignette followed the same structure, starting with an introduction in which we briefly described the scenario and initial situation. This was followed by the main section, in which the leader (lieutenant) delivered a speech to his followers (recruits), using either cognitive, affective, or behavioral leader communication techniques. For example, in the cognitive vignettes, the leader uses cognitive leader communication techniques such as *explaining*, asking *summary questions*, *making a proposition*, and *expressing moral conviction*. In the affective vignettes, the leader uses leader communication techniques such as *praising others*, *agreeing* and keeping *eye contact*, and in the behavioral vignettes, the leader uses leader communication techniques such as *setting goals* and *giving precise instructions*.

All vignettes were comparable across conditions, as they contained a similar number of (a) words, (b) sentences, and (c) leader communication techniques. The vignettes within the same scenario only differed between conditions in the type of leader communication technique used (cognitive, affective, or behavioral leader communication techniques).

### 3.2. Pre-Study 1: Testing the Initial Vignettes

For the initially developed vignettes, we tested the extent to which military recruits would perceive and recognize the different leader communication techniques that were included in the vignettes. To this end, we conducted a within-subjects online survey as a pre-study, presenting the vignettes in a written format. An additional aim of the pre-study was to find out whether all nine developed vignettes were comparable in their tone, meaning that the vignettes were perceived as similarly friendly.

#### 3.2.1. Sample

In total, 86 German-speaking members of the Swiss Armed Forces participated in this pre-study. The sample consisted of 83 male, 2 female, and 1 non-binary military recruits, of whom 63 were soldiers, 17 were sergeants, and 6 were lieutenants. They were between 18 and 25 (*M* = 20.25, *SD* = 1.14) years old. Participants received a monetary reward of CHF 10 (approximately USD 11), which conformed with the compensation guidelines of the Swiss Armed Forces for participating in studies. One participant was excluded from the sample because he did not correctly respond to attention check items, that is, he could not recall the name of the lieutenant in the vignettes and remembered only one of the topics of the three scenarios in the vignettes.

#### 3.2.2. Procedure and Measures

Each participant read all nine developed vignettes in a randomized order. After each vignette, participants indicated which leader communication techniques they recognized in the vignettes. They were asked to indicate whether they perceived the leader communication techniques described therein (“Below you can see three groups of different behaviors. For each group of behaviors, please indicate how they apply to the lieutenant’s previous speech”). In the first item, we listed the five cognitive leader communication techniques; in the second item, we listed the four affective leader communication techniques (we could not implement the fifth technique, eye contact, in the written version of the vignette); and in the third item, we listed the five behavioral leader communication techniques. Participants were then asked to rate each item on a 7-point Likert scale, ranging from 1 (*does not apply at all*) to 7 (*applies completely*).

In addition, participants rated the vignettes’ tone with a single item (“The lieutenant is friendly, polite, respectful, speaks appropriately”) on a 7-point Likert scale ranging from 1 = *does not apply at all* to 7 = *applies completely*. Answer checks were applied for the whole survey so that participants were only able to proceed to the next survey page if they answered all items.

#### 3.2.3. Results and Discussion

We computed an analysis of variance (ANOVA) with repeated measures and pairwise t-tests with Bonferroni correction to examine the extent to which participants recognized the leader communication techniques that were built into the vignettes. Results are shown in Table 2.

For the *cognitive* vignette of the scenario *room tidiness*, results showed that participants recognized the intended cognitive leader communication techniques that were present in the vignette (*M* = 5.38, *SD* = 1.17) significantly more often than the affective leader communication techniques that were not present in the vignette (*M* = 4.35, *SD* = 1.41), but they recognized the behavioral leader communication techniques that were also not present in the vignette (*M* = 5.29, *SD* = 1.20) just as frequently as the intended cognitive leader communication techniques (*F*(2, 170) = 25.45, *p* < 0.001, ges = 0.12). For the *affective* vignette of the same scenario, participants recognized the intended affective leader communication techniques (*M* = 5.80, *SD* = 1.21) significantly more often than the unintended cognitive (*M* = 4.91, *SD* = 1.33) and behavioral leader communication techniques (*M* = 4.38, *SD* = 1.47; *F*(1.64, 139.69) = 28.49, *p* < 0.001, ges = 0.16). Similarly, in the *behavioral* vignette of the same scenario, participants perceived the intended behavioral leader communication techniques (*M* = 6.08, *SD* = 1.01) significantly more often than the unintended cognitive (*M* = 5.06, *SD* = 1.42) and affective leader communication techniques (*M* = 4.11, *SD* = 1.48; *F*(2, 170) = 57.48, *p* < 0.001, ges = 0.28).

For the *cognitive* vignette of the scenario *marching drill*, results showed that participants recognized the intended cognitive leader communication techniques (*M* = 5.22, *SD* = 1.25) just as often as the unintended behavioral (*M* = 5.12, *SD* = 1.38) leader communication techniques, but they recognized the unintended affective leader communication techniques (*M* = 4.55, *SD* = 1.22) significantly less often than both the intended cognitive and unintended behavioral leader communication techniques (*F*(2, 170) = 8.94, *p* < 0.001, ges = 0.05). For the *affective* vignette of the same scenario, participants perceived the intended affective leader communication techniques (*M* = 5.79, *SD* = 1.19) significantly more often than the unintended cognitive (*M* = 4.97, *SD* = 1.30) and behavioral leader communication techniques (*M* = 4.45, *SD* = 1.25; *F*(2, 170) = 33.33, *p* < 0.001, ges = 0.17). Similarly, in the *behavioral* vignette of the same scenario, participants perceived the intended behavioral leader communication techniques (*M* = 5.78, *SD* = 1.07) significantly more often than the unintended cognitive (*M* = 5.15, *SD* = 1.25) and affective leader communication techniques (*M* = 4.45, *SD* = 1.49; *F*(1.85, 156.96) = 28.82, *p* < 0.001, ges = 0.15).

For the *cognitive* vignette of the scenario *voluntary continuation after basic military training*, results showed that participants recognized the intended cognitive leader communication techniques (*M* = 4.95, *SD* = 1.28) just as often as the unintended affective (*M* = 4.92, *SD* = 1.24) leader communication techniques, but they recognized the unintended behavioral leader communication techniques (*M* = 4.34, *SD* = 1.44) significantly less often than the intended cognitive and unintended affective leader communication techniques (*F*(1.86, 157.73) = 7.94, *p* < 0.001, ges = 0.04). For the *affective* vignette of the same scenario, participants perceived the intended affective leader communication techniques (*M* = 5.88, *SD* = 1.18) significantly more often than the unintended cognitive (*M* = 4.54, *SD* = 1.38) and behavioral leader communication techniques (*M* = 4.31, *SD* = 1.60; *F*(1.84, 156.59) = 38.56, *p* < 0.001, ges = 0.20). In the *behavioral* vignette of the same scenario, participants perceived the unintended affective (*M* = 5.41, *SD* = 1.31) leader communication techniques significantly more often than the intended behavioral leader communication techniques (*M* = 4.49, *SD* = 1.45), but they recognized the behavioral leader communication techniques just as often as the cognitive (*M* = 4.72, *SD* = 1.41) leader communication techniques (*F*(2, 170) = 13.06, *p* < 0.001, ges = 0.07).

In addition, we conducted an ANOVA with repeated measures to assess whether the vignettes were perceived similarly in their tone (see Table 3). Results showed that, for the *room tidiness* scenarios, the vignettes differed significantly in their tone (*F*(1.89, 160.56) = 17.67, *p* < 0.001, ges = 0.09). Pairwise t-tests with Bonferroni correction showed that the *affective* vignette (*M* = 6.02; *SD* = 1.16) was perceived significantly as friendlier than the *cognitive* (*M* = 5.66; *SD* = 1.00) and *behavioral* (*M* = 5.14; *SD* = 1.28) vignettes. For the *marching drills* scenarios, results were similar (*F*(2, 160) = 10.43, *p* < 0.001, ges = 0.06). The *affective* vignette (*M* = 5.91; *SD* = 1.09) was perceived as friendly as the *cognitive* vignette (*M* = 5.72; *SD* = 0.93) but significantly friendlier than the *behavioral* vignette (*M* = 5.39; *SD* = 1.00). For the scenario *voluntary continuation after basic military training*, the vignettes also differed significantly in their tone (*F*(2, 170) = 3.91, *p* = 0.02, ges = 0.02). The *affective* vignette (*M* = 6.02; *SD* = 0.96) was perceived as friendly as the *behavioral* vignette (*M* = 5.76; *SD* = 1.07) but significantly friendlier than the *cognitive* vignette (*M* = 5.70; *SD* = 1.10).

In summary, the results for testing the initially developed vignettes suggest that the participants were not fully able to accurately identify the leader communication techniques included in them. Additionally, the vignettes appeared to be different in their tone (i.e., level of friendliness). Thus, the initially developed vignettes required revision. We implemented three changes to improve the vignettes. First, we removed explanatory parts from the affective vignettes so that the explanatory aspects only appeared in the cognitive vignettes. Second, we revised the arguments in the cognitive vignette to make them more distinct from those in the affective vignettes. We achieved this by placing greater emphasis on explanation, inquiry, and moral conviction in the cognitive vignettes. Third, to ensure that the cognitive and behavioral vignettes were perceived as equally friendly as the affective vignettes, we adjusted the phrasing while keeping the content consistent. For instance, we used “I” messages in the behavioral vignettes instead of direct command formulation.

### 3.3. Pre-Study 2: Testing the Revised Vignettes

To test the revised written vignettes in terms of whether participants could now accurately recognize the leader communication techniques and whether all nine vignettes were comparable in tone (i.e., perceived as similarly friendly), we conducted a second pre-study with infantry recruits. We used a within-subjects design to test the nine vignettes.

#### 3.3.1. Sample

In total, 78 German-speaking military recruits from the infantry of the Swiss Armed Forces participated in this pre-study. The sample consisted of 77 male and 1 female recruits. They were between 19 and 25 (*M* = 20.15, *SD* = 1.21) years old. In return for their participation, participants received a monetary reward of CHF 10 (approximately USD 11), which is in accordance with the compensation guidelines of the Swiss Armed Forces for participating in research studies.

#### 3.3.2. Procedure and Measures

The procedure in the second pre-study was the same as in the first pre-study. Participants were again asked to indicate whether they perceived the leader communication techniques described therein (“Below you can see three groups of different behaviors. For each group of behaviors, please indicate the extent to which they apply to the lieutenant’s previous speech”). To make it easier for the participants to understand what we meant by the techniques, we did not list the names of the techniques in the items (e.g., the lieutenant asks control questions) but used descriptions (e.g., the lieutenant asks the recruits whether they have understood what they are supposed to do next). In the first item, we listed a description of the five cognitive leader communication techniques; in the second item, we listed a description of the four affective leader communication techniques (without the fifth technique, eye contact); and in the third item, we listed a description of the five behavioral leader communication techniques. Participants rated each item on a 7-point Likert scale, ranging from 1 (*does not apply at all*) to 7 (*applies completely*).

#### 3.3.3. Results and Discussion

We computed an ANOVA with repeated measures and pairwise t-tests with Bonferroni correction to examine the extent to which participants recognized the leader communication techniques that were built into the revised vignettes. The results suggest that the revision of the vignettes was successful in that the vignettes of each condition were clearly recognized as such (see Table 4). For the scenario *room tidiness*, results showed that in the *cognitive* vignette, participants recognized the intended cognitive leader communication techniques (*M* = 5.63, *SD* = 1.06) significantly more often than the unintended affective (*M* = 4.83, *SD* = 1.52) and behavioral leader communication techniques (*M* = 4.97, *SD* = 1.55; *F*(2, 154) = 10.81, *p* < 0.001, ges = 0.06). In the *affective* vignette of the same scenario, participants recognized the intended affective leader communication techniques (*M* = 5.65, *SD* = 1.26) significantly more often than the unintended cognitive (*M* = 5.14, *SD* = 1.31) and behavioral leader communication techniques (*M* = 4.39, *SD* = 1.61; *F*(1.84, 141.31) = 20.72, *p* < 0.001, ges = 0.12). In the *behavioral* vignette of the same scenario, participants recognized the intended behavioral leader communication techniques (*M* = 5.64, *SD* = 1.31) significantly more often than the unintended cognitive (*M* = 4.78, *SD* = 1.47) and affective leader communication techniques (*M* = 4.22, *SD* = 1.52; *F*(1.88, 144.99) = 28.40, *p* < 0.001, ges = 0.14).

For the scenario *marching* drills, results showed that in the *cognitive* vignette participants recognized the intended cognitive leader communication techniques (*M* = 5.54, *SD* = 1.10) significantly more often than the unintended affective (*M* = 4.83, *SD* = 1.26) and behavioral leader communication techniques (*M* = 4.92, *SD* = 1.49; *F*(2, 154) = 8.92, *p* < 0.001, ges = 0.06). In the *affective* vignette of the same scenario, participants recognized the intended affective leader communication techniques (*M* = 5.65, *SD* = 1.31) significantly more often than the unintended cognitive (*M* = 4.59, *SD* = 1.37) and behavioral leader communication techniques (*M* = 4.47, *SD* = 1.47; *F*(1.87, 144.06) = 26.35, *p* < 0.001, ges = 0.13). In the *behavioral* vignette of the same scenario, participants recognized the intended behavioral leader communication techniques (*M* = 5.23, *SD* = 1.28) significantly more often than the unintended affective leader communication techniques (*M* = 4.05, *SD* = 1.63), but they recognized the behavioral leader communication techniques just as often as the cognitive leader communication techniques (*M* = 4.87, *SD* = 1.30; *F*(1.80, 138.34) = 18.02, *p* < 0.001, ges = 0.11).

For the scenario *voluntary continuation after basic military training*, results showed that in the *cognitive* vignette, participants recognized the intended cognitive leader communication techniques (*M* = 4.92, *SD* = 1.31) just as often as the unintended affective (*M* = 4.73, *SD* = 1.37) and behavioral leader communication techniques (*M* = 4.59, *SD* = 1.52; *F*(2, 154) = 1.53, *p* = 0.22, ges = 0.01). In the *affective* vignette of the same scenario, participants recognized the intended affective leader communication techniques (*M* = 5.82, *SD* = 1.23) significantly more often than the unintended cognitive (*M* = 4.64, *SD* = 1.66) and behavioral leader communication techniques (*M* = 4.58, *SD* = 1.59; *F*(2, 154) = 23.54, *p* < 0.001, ges = 0.13). In the *behavioral* vignette of the same scenario, participants recognized the intended behavioral leader communication techniques (*M* = 5.44, *SD* = 1.34) significantly more often than the unintended cognitive (*M* = 4.71, *SD* = 1.55) and affective leader communication techniques (*M* = 4.58, *SD* = 1.45; *F*(2, 154) = 9.49, *p* < 0.001, ges = 0.07).

We conducted ANOVA with repeated measures to assess whether the vignettes were perceived as similar in their tone (see Table 5). Pairwise t-tests with Bonferroni correction showed that for the scenario *room tidiness*, participants perceived the *behavioral* vignette (*M* = 5.38; *SD* = 1.09) as significantly less friendly than the affective vignette (*M* = 5.86; *SD* = 1.04) but just as friendly as the cognitive (*M* = 5.56; *SD* = 1.19) vignette (*F*(2, 140) = 5.99, *p* = 0.003, ges = 0.04). Similarly, for the scenario *marching drills*, results showed that participants perceived the *behavioral* vignette (*M* = 5.01; *SD* = 1.18) as significantly less friendly than the affective (*M* = 5.95; *SD* = 1.01) and cognitive (*M* = 5.49; *SD* = 1.05) vignettes. Participants perceived the cognitive vignette as significantly less friendly than the affective vignette (*F*(2, 142) = 17.36, *p* < 0.001, ges = 0.11). For the scenario *voluntary continuation after basic military training*, results showed that participants perceived the affective vignette (*M* = 6.13; *SD* = 0.88) as significantly friendlier than the cognitive (*M* = 5.61; *SD* = 1.02) and behavioral (*M* = 5.49; *SD* = 1.06) vignettes (*F*(2, 144) = 12.91 *p* < 0.001, ges = 0.08).

In summary, the results for testing the revised vignettes suggest that for most vignettes, participants were able to recognize the leader communication techniques that were included in the vignettes. The two vignettes that required revision were the behavioral vignette of the scenario “marching drills” and the cognitive vignette of the scenario “voluntary continuation after basic military training”. Here, participants were not fully able to accurately identify the leader communication techniques included.

To make these vignettes more distinct, we increased the behavioral aspects and removed the cognitive aspects in the behavioral vignette and increased the cognitive aspects in the cognitive vignette. Specifically, we reduced the focus on information and explanation in the behavioral vignette and increased it in the cognitive vignette. Conversely, in the behavioral vignette, we augmented elements pertaining to instructions and goal setting. We implemented these changes primarily by changing the phrasing while keeping the content consistent. Regarding the tone of the vignettes, we decided not to change the content of the vignettes to avoid influencing the results in terms of the perception of the leader’s communication techniques. The final written vignettes can be found in the Supplementary Materials (File S1).

### 3.4. Main Study: Experimental Testing of Video Vignettes

To test our hypotheses, we conducted a between-subjects experiment with three conditions: the use of (1) cognitive versus (2) affective versus (3) behavioral leader communication techniques. We manipulated leader communication techniques using video vignettes. Participants were randomly assigned to one of the three conditions, and they were unaware of their assigned condition. The study was preregistered (https://aspredicted.org/ndj2-wm3h.pdf, accessed on 18 June 2025).

#### 3.4.1. Sample

Participants were German-speaking recruits from different branches of service of the Swiss Armed Forces. A total of 377 recruits participated voluntarily in this study. We excluded one participant from the data set due to responding to all items without any variations (i.e., at the lowest scale point 1). Furthermore, we excluded four participants because they did not respond correctly to the attention check items, that is, they could not recall the name of the lieutenant and remembered only one or none of the three scenario topics. We also excluded four participants who indicated that their native language was not German and that they lacked proficiency in the German language.

The final sample consisted of 368 recruits (male = 358, female = 8, and non-binary = 2) from different branches of service (maintenance = 149, air defense = 113, and infantry = 106). They were between 18 and 32 years old (*M* = 20.20, *SD* = 1.59). Regarding participants’ education, 226 participants had a certified apprenticeship, 49 had an additional professional baccalaureate, 73 had completed upper secondary school, three had a degree in applied sciences, seven had a university degree, and 10 indicated that they had another level of education. Similar to the pre-studies, participants received a monetary reward of CHF 10 (approximately USD 11) as compensation for their participation in the study, which conforms with the compensation guidelines of the Swiss Armed Forces for participating in studies.

#### 3.4.2. Video Vignettes

For this study, we used the (partly revised) vignettes from the second pre-test (pre-study 2), which were professionally recorded on video in a studio setting. The role of the lieutenant in the vignettes was performed by an experienced actor who also served in the Swiss Armed Forces and was therefore familiar with the relevant military etiquette. Subsequently, the videos were approved by a subject matter expert (i.e., a prospective career officer) for the purpose of ensuring that the videos and the lieutenant were realistic.

Every video vignette started with a brief introduction, asking participants to imagine that the lieutenant called the participants and their comrades over to talk to them about a specific issue. This introductory part was followed by the main part of the vignettes. As described earlier, the vignettes presented three different scenarios on topics relevant to the participants (i.e., room tidiness, military marching drills, or voluntary continuation after basic military training). Participants watched three vignettes corresponding to their assigned condition (cognitive, affective, or behavioral). For example, in the cognitive conditions, the leader used only cognitive leader communication techniques in each of the three scenarios, whereas in the affective condition, the leader used only affective leader communication techniques in each of the three scenarios.

We ensured that the vignettes in each condition were of comparable length, using the same number of techniques. They matched in word and sentence count and incorporated the same leader communication techniques across all scenarios within the same condition.

#### 3.4.3. Procedure

The first author of this paper welcomed and informed the participants about the research project and the study procedure. Participants were asked to give their informed consent to participate in this study and could then start with the experiment on the computer. Each participant was randomly assigned to one of the three conditions (cognitive, *n* = 126; affective, *n* = 119; behavioral, *n* = 123) and watched three video vignettes on their individual computer screen. The three vignettes (room tidiness, marching drills, and voluntary continuation after military basic training) within each condition were displayed in a randomized order.

After participants finished watching the first video vignette, they completed a survey assessing proximal follower outcomes as reactions to the (cognitive, affective, or behavioral) vignette they had just been presented with. Participants completed the same survey assessing proximal follower outcomes for the second and third vignettes, too.

After participants had watched all three video vignettes of their condition, they completed a survey assessing distal follower outcomes, manipulation check items, and control variables. Answer checks were applied for all surveys so that participants were only able to proceed to the next survey page if they answered all items.

#### 3.4.4. Measures

Outcome variables were selected based on an in-depth literature review of leader communication effects (e.g., Antonakis et al., 2022; Boies et al., 2015). While the literature highlights a wide range of potential follower outcomes, we focused on a subset that aligns closely with the mechanisms theorized to be influenced by leader communication techniques. Participants rated the items of all outcomes and additional variables on a seven-point Likert scale ranging from 1 = *does not apply at all* to 7 = *applies completely*.

##### Proximal Cognitive Outcomes

Followers’ Task Understanding. We measured followers’ task understanding with three self-designed items. The items were: “With this lieutenant, I understand the purpose of this task”, “With this lieutenant, I understand the reasons behind this task”, and “With this lieutenant, I understand why this task is important”. Cronbach’s alpha was 0.89.

Followers’ Perception of Task Relevance. We assessed followers’ perceived task relevance in each scenario using a single item per scenario. The item was “This task is relevant”.

Followers’ Lack of Role Ambiguity. We assessed the lack of role ambiguity with three items based on the Lack of Role Ambiguity Scale from Rizzo et al. (1970) that we adapted to the military context. An example of an item is: “With this lieutenant, I know exactly what I have to do”. Cronbach’s alpha was 0.89. The allocation of this variable to the cognitive follower outcomes differs from the pre-registration. We decided to change the variable *lack of role ambiguity* from the behavioral to cognitive follower outcome because role ambiguity has more cognitive than behavioral aspects.

Leader Clarity. We measured followers’ perception of leader clarity with four items, similar to prior studies (e.g., Abele et al., 2008; Antonakis et al., 2011). An example of an item is: “This lieutenant seems to be determined”. Cronbach’s alpha was 0.90.

Leader Competence. We measured followers’ perception of leader competence with four items based on previous research (e.g., Abele et al., 2008, 2016; Carrier et al., 2014; Fiske et al., 2002). An example item is: “The lieutenant seems competent”. Cronbach’s alpha was 0.91.

##### Distal Cognitive Outcomes

Followers’ Occupational Self-Efficacy. Followers’ occupational self-efficacy was assessed using a short version of the occupational self-efficacy scale (Rigotti et al., 2008). From the short version of the scale, we used the three items with the highest factor loadings. An example item is: “If this lieutenant was my leader, I would face difficulties calmly, because I could always rely on my abilities”. Cronbach’s alpha was 0.83.

Followers’ Attitudes Toward Job Tasks. We measured followers’ expected change in attitudes toward relevant job tasks with one item per scenario. The item was: “If the lieutenant were my platoon leader, my attitude towards room tidiness (marching drills or voluntary continuation after basic military training) would improve”.

Leader Endorsement. We measured the extent to which participants endorsed the leader using three items adapted from Ullrich et al. (2009). An example item is: “This lieutenant would be the right person for this leadership role”. Cronbach’s alpha for the scale was 0.86.

Leader Persuasiveness. We measured leader persuasiveness with two self-designed items, similar to prior leadership studies (e.g., Platow et al., 2006). The items used to measure followers’ persuasiveness were: “This lieutenant would have a positive influence on me as a leader” and “This lieutenant would influence my thinking”. Cronbach’s alpha was 0.75.

##### Proximal Affective Outcomes

Followers’ Task Motivation. We assessed whether followers were motivated for the task, similar to prior studies (e.g., Boekaerts, 2002; Poupore, 2016; Wang, 2016). We used two items to measure the extent to which the participants felt that they would enjoy the task and be motivated for it. An example item is: “I am motivated for this task”. Cronbach’s alpha was 0.79.

Followers’ Sense of Belonging. We assessed followers’ sense of belonging, similar to other leadership studies (e.g., Good et al., 2012; Hoogervorst et al., 2012; Ruggieri & Abbate, 2013). We used three items to measure the extent to which the participants felt that they would be accepted, respected, and included. An example item is: “With the lieutenant, I feel respected”. Cronbach’s alpha was 0.84.

Leader Warmth. We measured to what extent participants felt that the leader appeared to be kind, content, and trustworthy using six items based on prior literature (Abele et al., 2008, 2016; Carrier et al., 2014; Fiske et al., 2002; A. C. Little et al., 2012). An example item is: “This lieutenant is trustworthy”. Cronbach’s alpha was 0.92.

##### Distal Affective Outcomes

Followers’ Occupational Self-Esteem. We assessed followers’ self-esteem with the Organizational Self-Esteem Scale (Kanning & Hill, 2012). We used the three items with the highest factor loadings. An example item is: “If this lieutenant was my leader, I would feel that people believed in me”. Cronbach’s alpha was 0.92.

Followers’ Affective Organizational Commitment. To measure followers’ affective organizational commitment, we used the three items with the highest factor loadings of the Affective Organizational Commitment Scale by Felfe et al. (2002). An example item is: “If this lieutenant was my leader, I would be proud to belong to the Swiss Armed Forces”. Cronbach’s alpha was 0.78.

Leader Liking. We measured whether participants liked the leader with a single-item measure (Antonakis et al., 2011). The item was: “I like this lieutenant as a leader”.

Leader Charisma. We assessed leaders’ charisma with three items based on Grabo and van Vugt (2016). An example item is: “This lieutenant is inspiring”. Cronbach’s alpha was 0.86.

##### Proximal Behavioral Outcomes

Followers’ Behavioral Intention to Perform a Task. We measured followers’ intentions to perform the task (i.e., the scenarios of the vignettes), similar to other studies measuring behavioral intentions (e.g., Lyons et al., 2009; Mayfield & Mayfield, 2007; Sirois, 2004), with two items. An example item is: “With this lieutenant, I would adhere with the room rules in an appropriate manner”. Cronbach’s alpha was 0.81.

##### Distal Behavioral Outcomes

Followers’ Task Performance. We measured followers’ task performance using the three items with the highest factor loadings from the In-Role Behavior Scale by Williams and Anderson (1991), which we adapted to the military context. An example item was “If this lieutenant was my leader, I would fulfill my orders in an appropriate manner”. Cronbach’s alpha was 0.86.

Followers’ Intention to Change Behavior. We measured followers’ intention to change their behavior related to the vignettes with a single item per scenario. The item was: “If this lieutenant was my leader, I would always keep the room tidy until the end of the basic military training”, adapted from Pai and Edington (2008).

Leader Following. To assess whether participants would actually follow the lieutenant from the vignettes, we used two self-designed items: “I would want to follow this lieutenant” and “I would obey this lieutenant”. Cronbach’s alpha was 0.65.

##### Control Variables

Attitudes toward Armed Forces. We assessed participants’ attitudes toward the armed forces with the three items with the highest factor loadings from a scale from Blaser and Signorell (2008). An example item is: “I joined the basic military training with positive expectations”. Cronbach’s alpha was 0.83.

Control variables. We additionally assessed three control items to check whether there were any notable differences between the participants in the three conditions with regard to these variables. In the military, it is very important to present oneself correctly and to wear a neat uniform. We wanted to determine whether the participants perceived this in the lieutenant (regardless of the lack of insignia related to function and branch of service, which we deliberately omitted to avoid the influence of possible stereotypes). We therefore controlled for this with one item: “Apart from the two missing insignia (function and branch of service), the lieutenant’s uniform was correct”. We also wanted to assess how strongly the participants judged the lieutenants’ nonverbal communication and whether they judged the nonverbal behavior to be consistent with the content. The items for this were: “How did you perceive the lieutenant’s nonverbal communication (body language)?” and “The lieutenant’s facial expressions and gestures (hand and arm movements) were consistent with what he was saying”.

## 4. Results

### 4.1. Preliminary Analyses

As a preliminary analysis, we tested whether the participants in the three conditions differed from each other by chance. For this, we computed ANOVAs with pairwise t-tests with Bonferroni correction. We did not find any differences in participants’ attitudes toward the armed forces. Participants in the cognitive condition (*M* = 4.32, *SD* = 1.33) had similar attitudes toward the armed forces as participants in the affective (*M* = 4.28, *SD* = 1.42) and behavioral (*M* = 4.22, *SD* = 1.23) conditions (*F*(2, 365) = 0.18, *p* = 0.84, η^2^ < 0.001).

Regarding the assessed control variables, results showed that participants in the cognitive (*M* = 4.98, *SD* = 1.57), affective (*M* = 4.92, *SD* = 1.69), and behavioral (*M* = 4.87, *SD* = 1.64) conditions did not differ in their perceptions of the lieutenant’s uniform (*F*(2, 365) = 0.16, *p* = 0.86, η^2^ < 0.001). Furthermore, results showed that participants in the cognitive (*M* = 4.54, *SD* = 1.10), affective (*M* = 4.80, *SD* = 1.16), and behavioral (*M* = 4.51, *SD* = 1.19) conditions did not judge the lieutenant’s nonverbal communication differently (*F*(2, 354) = 2.20, *p* = 0.11, η^2^ = 0.01). Regarding the congruence between the lieutenant’s non-verbal communication and the content of his speech, results indicated that participants in the cognitive (*M* = 4.85, *SD* = 1.21), affective (*M* = 4.92, *SD* = 1.41), and behavioral (*M* = 4.72, *SD* = 1.36) conditions perceived the lieutenant as congruent. In addition, the participants did not differ significantly in their assessment of congruence (*F*(2, 365) = 0.71, *p* = 0.49, η^2^ < 0.01). Since there were no differences between the cognitive, affective, and behavioral conditions with respect to these control variables, we proceeded with the analyses.

We conducted confirmatory factor analyses (CFAs) to check that all cognitive, affective, and behavioral outcome variables were sufficiently distinct from each other. We found that the cognitive outcome variables *leader clarity* and *leader competence* showed a very high latent correlation (0.98). We therefore decided to merge these two variables into a single factor labeled *leader competence*. The fit indices of the final CFAs for the cognitive (CFI = 0.94; RMSEA = 0.07; SRMR = 0.05), affective (CFI = 0.94; RMSEA = 0.07; SRMR = 0.06), and behavioral (CFI = 0.98; RMSEA = 0.08; SRMR = 0.02) follower outcomes indicated acceptable fit and showed that the remaining follower outcomes were distinct. All factor loadings for the cognitive outcomes ranged from 0.73 to 1.00 (*Mdn* = 0.85). For the affective outcomes, factor loadings ranged from 0.41 to 1.00 (*Mdn* = 0.88), and for the behavioral outcomes, factor loadings ranged from 0.69 to 1.00 (*Mdn* = 0.85). Latent factor intercorrelations were below 0.91 for the remaining cognitive outcomes (*Mdn* = 0.55), below 0.72 for the affective outcomes (*Mdn* = 0.44), and below 0.84 for the behavioral outcomes (*Mdn* = 0.68). The fit indices for the CFA with all outcome variables (CFI = 0.90; RMSEA = 0.07; SRMR = 0.06) indicated acceptable fit and showed that the follower outcomes were distinct. All factor loadings ranged from 0.41 to 1.00 (*Mdn* = 0.86). Latent factor intercorrelations were below 0.91 (*Mdn* = 0.54).

We did not find significant differences in the proximal outcomes between the three scenarios within each condition. Therefore, we averaged the ratings of the proximal outcomes across the three scenarios (e.g., task motivation for room tidiness, task motivation for marching drills, and task motivation for voluntary continuation after basic military training) to calculate the score for an overall proximal outcome (e.g., task motivation). The distal outcomes were assessed only once, after the video vignettes of all three scenarios had been watched by the participants (in accordance with our definition of distal outcomes).

### 4.2. Hypothesis Testing

We computed multivariate analyses of variance (MANOVAs) to examine whether cognitive versus affective versus behavioral leader communication techniques influenced participants’ cognitive, affective, versus behavioral follower outcomes. We computed a MANOVA for every category of outcomes, and we used the conditions (cognitive vs. affective vs. versus behavioral) as between-subjects factors. Table 6, Table 7 and Table 8 present the means, standard deviations, and correlations of all study variables per condition. We performed all the calculations using the software R (Version 2024.09.1+394, R Core Team, 2024).

Hypothesis 1 stated that the cognitive leader communication techniques would have a positive influence on cognitive follower outcomes. The multivariate test using the test statistic Wilks’ Lambda yielded significant differences in the proximal cognitive follower outcomes (*Λ* = 0.95, *F*(8, 724) = 2.46, *p* = 0.01). Post-hoc tests for subsequent univariate analyses, however, showed no significant mean differences in followers’ task understanding (*F*(2, 365) = 1.45, *p* = 0.24, η^2^ < 0.01), followers’ perception of task relevance (*F*(2, 365) = 1.47, *p* = 0.23, η^2^ < 0.01), followers’ lack of role ambiguity (*F*(2, 365) = 1.74, *p* = 0.18, η^2^ = 0.01), and leader competence (*F*(2, 365) = 0.03, *p* = 0.97, η^2^ < 0.001).

For the distal cognitive follower outcomes, the MANOVA results were not significant, indicating that there were no significant mean differences in distal cognitive follower outcomes between the cognitive, affective, and behavioral vignettes (*Λ* = 0.98, *F*(8, 724) = 0.95, *p* = 0.48). Results of subsequent univariate analyses showed no significant mean differences in followers’ occupational self-efficacy (*F*(2, 365) = 0.19, *p* = 0.82, η^2^ < 0.01), followers’ attitudes toward job tasks (*F*(2, 365) = 2.13, *p* = 0.12, η^2^ = 0.01), leader endorsement (*F*(2, 365) = 0.04, *p* = 0.97, η^2^ < 0.001), and leader persuasiveness (*F*(2, 365) = 0.56, *p* = 0.58, η^2^ < 0.01).

Hypothesis 2 stated that the affective leader communication techniques would have a positive influence on affective follower outcomes. The multivariate test using the test statistic Wilks’ Lambda yielded a significant difference in the proximal affective follower outcomes (*Λ* = 0.87, *F*(6, 718) = 8.75, *p* < 0.001). Post-hoc tests for subsequent univariate analyses showed a significant mean difference in leader warmth (*F*(2, 363) = 19.96, *p* < 0.001, η^2^ = 0.10). More specifically, results showed that participants felt that the lieutenant was warmer in the affective vignette (*M* = 5.59, *SD* = 0.72) than in the cognitive (*M* = 5.22, *SD* = 0.75) and behavioral vignette (*M* = 4.95, *SD* = 0.87). In contrast, post-hoc tests showed no significant mean difference in followers’ task motivation (*F*(2, 365) = 0.05, *p* = 0.95, η^2^ < 0.001) and followers’ sense of belonging (*F*(2, 363) = 0.04, *p* = 0.96, η^2^ < 0.001).

For the distal affective follower outcomes, the MANOVA results were not significant, indicating that there were no significant mean differences in distal affective follower outcomes (*Λ* = 0.96, *F*(8, 700) = 1.94, *p* = 0.05) between the cognitive, affective, and behavioral vignettes. The post-hoc tests for subsequent univariate analyses showed a significant mean difference in leader charisma (*F*(2, 365) = 4.33, *p* = 0.01, η^2^ = 0.02). More specifically, results showed that participants assessed the lieutenant as significantly more charismatic in the affective condition (*M* = 5.01, *SD* = 1.24) than in the behavioral condition (*M* = 4.53, *SD* = 1.29) but similarly charismatic as in the cognitive condition (*M* = 4.73, *SD* = 1.31). In contrast, post-hoc tests for subsequent univariate analyses showed no significant mean difference in followers’ occupational self-esteem (*F*(2, 353) = 0.80, *p* = 0.45, η^2^ < 0.01), affective organizational commitment (*F*(2, 365) = 2.36, *p* = 0.10, η^2^ = 0.01), and leader liking (*F*(2, 365) = 0.85, *p* = 0.43, η^2^ < 0.01).

Furthermore, our findings revealed a significant correlation between the affective outcomes of leader warmth and charisma and other follower outcomes. We found a significant correlation between leader warmth and leader competence in the cognitive (*r* = 0.73, *p* < 0.01), affective (*r* = 0.73, *p* < 0.01), and behavioral (*r* = 0.72, *p* < 0.01) conditions. Results further revealed a significant correlation between leader charisma and leader endorsement in the cognitive (*r* = 0.72, *p* < 0.01), affective (*r* = 0.71, *p* < 0.01), and behavioral (*r* = 0.75, *p* < 0.01) conditions. We also found a significant correlation between leader charisma and leader liking in the cognitive (*r* = 0.64, *p* < 0.01), affective (*r* = 0.72, *p* < 0.01), and behavioral condition (*r* = 0.65, *p* < 0.01).

Hypothesis 3 stated that the behavioral leader communication techniques would have a positive influence on behavioral follower outcomes. ANOVA results indicated no significant mean differences in followers’ behavioral intention to perform a task (*F*(2, 365) = 0.52, *p* = 0.59, η^2^ < 0.01).

For the distal behavioral follower outcomes, results were again not significant, indicating that there were no significant mean differences in distal behavioral follower outcomes (*Λ* = 0.99, *F*(6, 720) = 0.71, *p* = 0.64) between the cognitive, affective, and behavioral vignettes. Post-hoc tests for subsequent univariate analyses showed no significant mean difference in followers’ task performance (*F*(2, 362) = 0.32, *p* = 0.73, η^2^ < 0.01), followers’ long-term intention to change behavior (*F*(2, 365) = 1.64, *p* = 0.20, η^2^ = 0.01), and leader following (*F*(2, 365) = 0.31, *p* = 0.73, η^2^ < 0.01).

## 5. Discussion

There is consensus in leadership research that effective leadership requires good communication from the leader (Antonakis et al., 2016; Bass, 1985; Yukl et al., 2002). Despite this consensus, research on leader communication techniques remains highly fragmented, as many studies only investigate the effectiveness of single communication techniques (e.g., asking questions; Meyer et al., 2016). To enable research that systematically compares the effectiveness of different leader communication techniques, the present study developed a framework that distinguishes between cognitive, affective, and behavioral leader communication techniques based on a literature review and subject matter expert ratings. To gain knowledge about the relative effectiveness of the identified communication techniques, we conducted a vignette experiment that compared the effects of cognitive, affective, and behavioral leader communication techniques on various follower outcomes while keeping all other characteristics of the leader and their communication constant.

### 5.1. Key Findings and Theoretical Implications

A first key finding is that subject matter experts can distinguish between cognitive, affective, and behavioral leader communication techniques. We found that verbal communication techniques can be assigned to one of these categories, whereas para- and nonverbal communication techniques could hardly be assigned to these categories. We further found that subject matter experts judged verbal leader communication techniques generally as more effective than para- and nonverbal leader communication techniques. Taken together, this implies that it makes sense for research to prioritize studying verbal communication techniques (e.g., Antonakis et al., 2022; Hemshorn de Sanchez et al., 2024; Van Quaquebeke & Felps, 2018; Weiss et al., 2018) and that the categorization of cognitive, affective, and behavioral (verbal) communication techniques appears to be a fruitful approach to integrate the fragmented body of research on leader communication.

A second key finding is that—if everything else is held constant, including all leader attributes (demographics, appearance, personality, etc.), the content and length of the leaders’ communication, and even the tone/friendliness of the message—leader communication techniques do not differ much in their effectiveness. Specifically, we found no significant mean differences between the cognitive, affective, and behavioral conditions in all follower outcomes except for leader warmth and leader charisma. The absence of significant mean differences likely reflects several important considerations about how leader communication operates in practice. Prior studies on communication techniques for leaders have either investigated the communication techniques exhibited by different leaders (e.g., Meyer et al., 2016; Weiss et al., 2018) or have significantly altered the tone/friendliness of the conditions (e.g., Antonakis et al., 2022). While our controlled design successfully disentangles leader communication techniques from person effects and message tone, this very control may have revealed that specific communication techniques have limited impact when leaders maintain consistent friendliness. This suggests that followers may be more responsive to overall relational quality than to specific communication strategies, or that communication techniques require sustained interaction rather than brief exposure to demonstrate their effectiveness.

A third key finding is that, if one category of leader communication techniques is more effective than others, then it is the affective leader communication techniques. We found that participants whose leader used affective leader communication techniques felt that their leader was warmer compared to followers whose leader used cognitive or behavioral leader communication techniques. In addition, leader charisma was found to be significantly higher among participants whose leaders used cognitive and affective leader communication techniques compared to followers whose leaders used behavioral leader communication techniques. On a theoretical level, this finding can be interpreted in line with an evolutionary perspective. Emotions are of great importance, as they have central functions (functional perspective on emotions, e.g., Scherer, 1982). Emotions help us understand the relevance of stimuli and events in relation to our needs, plans, and goals, and therefore, they have an informative function. Accordingly, research has demonstrated that emotionally charged stimuli have a tendency to capture our attention (e.g., Yiend, 2010). Emotions also serve a social function by communicating our emotional state to others in our social environment (Keltner & Haidt, 1999; Scherer, 1982). As such, affective communication techniques may generate more attention due to their emotional component and provide more information about the leader, task, and relationship between the leader and follower. This could make them more effective than cognitive and behavioral leader communication techniques. This suggests that these techniques could be more effective than cognitive or behavioral leader communication techniques, reinforcing the importance of incorporating affective communication behaviors into leadership theories. Such integration aligns with leadership research that underscores the role of emotions and affective processes (Ashkanasy & Humphrey, 2011; L. M. Little et al., 2016).

### 5.2. Limitations

First, our experimental vignette study design minimized the impacts of potential confounding variables but may have limited external validity. Although we used realistic military scenarios to enhance relevance, the utilized scenarios could still somewhat differ from real-life situations and behavior (Aguinis & Bradley, 2014; Atzmüller & Steiner, 2010).

Second, we conducted this study in the military context. Even though this is a common setting for leadership studies (e.g., LePine et al., 2016; Sefidan et al., 2021), the military context is highly specific, particularly in terms of sample distribution (i.e., gender diversity) and communication dynamics. Our sample consisted mainly of male participants due to compulsory military service for men (Federal authorities of the Swiss confederation, 2024). This gender distribution is rarely found in work contexts outside the military. Moreover, communication in the military is formal, top-down, and often command-driven, reflecting a high power distance culture (Soeters et al., 2006). These hierarchical structures in the military might shape implicit communication norms (Hartel & Blascovich, 2008), and thus, followers’ expectations on how leaders should communicate might differ within the military context versus other work contexts. These aspects can potentially limit the generalizability of our results to other work contexts. Limiting this concern, the Swiss Armed Forces has a system in which most members work in regular civilian jobs. This implies that the difference between the military and civilian leaders is less pronounced than in other countries (i.e., where they are military leaders full-time), which may contribute to a closer alignment in the expectations of leadership in both contexts.

### 5.3. Future Research

First, this study offers valuable insights into leader-to-follower communication. However, it focuses solely on unidirectional communication, where leaders communicate with followers without considering the interactive or reciprocal nature of leadership processes (Fairhurst & Connaughton, 2014; Güntner et al., 2020). Consequently, future research should explore bidirectional communication, where followers’ reactions and responses are integrated into the communication dynamics (Hemshorn de Sanchez et al., 2022). This would allow for a more comprehensive understanding of how leadership communication is adapted and shaped in response to follower input and how this reciprocal interaction impacts followers’ cognition, affect, and behavior.

Second, our study showed that, in day-to-day leadership situations, primarily affective leader communication techniques were effective for leader warmth and charisma. However, prior research has demonstrated that different scenarios necessitate different behaviors from leaders to influence followers (e.g., Jung et al., 2003; Oreg & Berson, 2011; Stam et al., 2018). Therefore, it would be beneficial to investigate the impact of leader communication techniques in different contexts, such as in crisis situations, change management, or innovation processes. Future studies could focus on identifying which leader communication techniques are most effective in which situations and whether the effectiveness of affective, cognitive, and behavioral leader communication techniques varies depending on the dynamics and requirements of the respective leadership situation.

### 5.4. Practical Implications

In terms of practical implications, we suggest that leaders may not necessarily need to distinguish between cognitive, affective, and behavioral leader communication techniques when communicating with followers. Instead, they should focus on using communication techniques deliberately and with purpose. If prioritization is necessary, we suggest using more affective leader communication techniques (e.g., maintaining eye contact, praising, expressing confidence that goals can be achieved), as these techniques are more likely to have an effect on affective follower outcomes.

This is relevant across a range of applied contexts. For example, in a corporate environment, a project manager launching a new project may combine expressing moral conviction about the project’s purpose (cognitive), praising team contributions (affective), and proposing next steps (behavioral) to foster both clarity and motivation. In a hospital setting, a senior nurse leading a shift change could ask clarifying questions and explain procedural updates (cognitive), praise staff for their work under pressure, and encourage proactive problem solving (affective).

These findings also carry implications for the development of leadership training programs. Rather than learning and practicing many different leader communication techniques and distinguishing between cognitive, affective, and behavioral leader communication techniques, leadership trainings can be made more effective by including (verbal and affective) leader communication techniques.

## 6. Conclusions

This study developed and tested a framework that categorizes leader communication techniques based on their intended impact on followers’ cognitions, affect, or behavior, as well as the communication channel used (verbal, para-verbal, or nonverbal). Subject matter experts were able to distinguish among these categories, particularly for verbal techniques, which were also rated as generally more effective than para- and nonverbal techniques. Using an experimental video vignette design, we found that cognitive, affective, and behavioral leader communication techniques did not lead to significant differences across most follower outcomes when leader attributes and message tone were held constant—except for leader warmth and charisma. Affective leader communication techniques were associated with higher perceptions of leader warmth and, together with cognitive techniques, with higher ratings of leader charisma. These findings suggest that affective communication techniques may be particularly relevant for shaping positive emotions in followers. The study contributes to integrating a previously fragmented research landscape on leader communication and provides a basis for future research on the effectiveness of leader communication techniques.

## Figures and Tables

**Table 1:** Overview of verbal, paraverbal, and non-verbal leader communication techniques with efficacy over 3.5.

	Communication Channel	Verbal = Linguistic Elements	Para-Verbal = All Aspects of the Voice	Nonverbal = All Forms of Movement of the Body and Face, as well as Body Position
Follower Impact				
**Cognitive =** leader communication techniques intended to influence followers’ thoughts, perception, memory, and learning		Asking questions Suggestion question (4.00) **Confirmation request (4.00)** Asking open questions (3.86) **Information request (4.00)** **Summary question (4.71)** Formulate a statement **Explaining (4.43)** **Expressing moral conviction (4.14)** Three-part lists (3.50)		
**Affective =** leader communication techniques intended to influence followers’ emotions and strengthen leader–follower relationship		Formulate a statement **Agreeing (4.71)** **Praising others (4.86)** **Expressing confidence that goals can be achieved (4.71)** Personal pronouns (4.00) Speaking in pictures (4.14) Speaking with stories (4.29) Formulating an imperative sentence **Encouragement (4.43)**	Animated voice tone Variation in loudness (3.71) Intended pauses (3.57) Word repetition (3.50)	Facial expressions **Eye contact (4.86)** Intensity of facial expressions (4.43) Gestures Body gestures (3.86) Head position (4.00) Smiling (3.86)
**Behavioral =** leader communication techniques intended to influence followers’ behavior		Asking questions **Control question (4.29)** Alternative question (3.71) Formulate a statement **Giving precise instructions (5.00)** Contrasts (3.57) **Making a proposition (4.71)** **Setting goals (4.86)** Formulate an imperative sentence **Command (4.57)**		

*Note.* Experts rated how effective they consider each leader communication technique to be in influencing followers’ cognitions, affect, and behavior using a scale from 1 (ineffective) to 5 (effective). The listed techniques have an average effectiveness assessment of more than 3.5. Mean score of effectiveness is indicated in brackets. Techniques printed in bold are among the five highest effective techniques. Some techniques are listed, for example, in the cognitive category despite having higher mean levels in the affective category (e.g., expressing moral conviction) because selection was based on within-category effectiveness rankings at the time of vignette development, irrespective of how those techniques scored across other categories (see chapter vignette structure).

**Table 2:** Overview of results of pre-study 1.

Vignette	Leader Communication Techniques	*M* (*SD*)	*DF*	*F*	*η^2^_p_*	*M*_Diff_
Room tidiness—cognitive scenario	Cognitive	5.38 (1.17)	2, 170	25.45 **	0.12	c-a = 6.73 **
	Affective	4.35 (1.41)				a-b = −5.28 **
	Behavioral	5.29 (1.20)				c-b = 0.63
Room tidiness—affective scenario	Cognitive	4.91 (1.33)	1.64, 139.69	28.49 **	0.16	c-a = −4.92 **
	Affective	5.80 (1.21)				a-b = 6.18 **
	Behavioral	4.38 (1.47)				c-b = 3.48 **
Room tidiness—behavioral scenario	Cognitive	5.06 (1.42)	2, 170	57.48 **	0.28	c-a = 5.52 **
	Affective	4.11 (1.48)				a-b = −9.60 **
	Behavioral	6.08 (1.01)				c-b = −5.93 **
Marching drills—cognitive scenario	Cognitive	5.22 (1.25)	2, 170	8.94 **	0.05	c-a = 3.88 **
	Affective	4.55 (1.22)				a-b = −3.39 **
	Behavioral	5.12 (1.38)				c-b = 0.61
Marching drills—affective scenario	Cognitive	4.97 (1.30)	2, 170	33.33 **	0.17	c-a = −4.89 **
	Affective	5.79 (1.19)				a-b = 7.94 **
	Behavioral	4.45 (1.25)				c-b = 3.23 **
Marching drills—behavioral scenario	Cognitive	5.15 (1.25)	1.85, 156.96	28.82 **	0.15	c-a = 4.66 **
	Affective	4.45 (1.49)				a-b = −6.67 **
	Behavioral	5.78 (1.07)				c-b = −3.65 **
Voluntary continuation—cognitive scenario	Cognitive	4.95 (1.28)	1.86, 157.73	7.94 **	0.04	c-a = 0.23
	Affective	4.92 (1.24)				a-b = 2.95 *
	Behavioral	4.34 (1.44)				c-b = 3.62 **
Voluntary continuation—affective scenario	Cognitive	4.54 (1.38)	1.84, 156.59	38.56 **	0.20	c-a = −7.93 **
	Affective	5.88 (1.18)				a-b = 7.06 **
	Behavioral	4.31 (1.60)				c-b = 1.20
Voluntary continuation—behavioral scenario	Cognitive	4.72 (1.41)	2, 170	13.06 **	0.07	c-a = −3.61 **
	Affective	5.41 (1.31)				a-b = 4.76 **
	Behavioral	4.49 (1.45)				c-b = 1.31

*Note.* * = *p* < 0.05; ** = *p* < 0.01.

**Table 3:** Overview of the results on comparing the vignettes’ tone in pre-study 1.

Scenario	Vignette	*M* (*SD*)	*DF*	*F*	*η^2^_p_*	*M*_Diff_
Room tidiness	Cognitive	5.66 (1.00)	1.89, 160.56	17.67 **	0.09	c-a = −2.68 *
	Affective	6.02 (1.16)				a-b = 5.23 **
	Behavioral	5.14 (1.28)				c-b = 3.66 **
Marching drills	Cognitive	5.72 (0.93)	2, 160	10.43 **	0.06	c-a = −1.59
	Affective	5.91 (1.09)				a-b = 4.32 **
	Behavioral	5.39 (1.00)				c-b = 2.92 *
Voluntary continuation	Cognitive	5.70 (1.10)	2, 170	3.91 *	0.02	c-a = −2.77 *
	Affective	6.02 (0.96)				a-b = 1.96
	Behavioral	5.76 (1.07)				c-b = −0.50

*Note*. * = *p* < 0.05; ** = *p* < 0.01.

**Table 4:** Overview of results of pre-study 2.

Vignette	Leader Communication Techniques	*M* (*SD*)	*DF*	*F*	*η^2^_p_*	*M*_Diff_
Room tidiness–cognitive scenario	Cognitive	5.63 (1.06)	2, 154	10.81 **	0.06	c-a = 4.58 **
	Affective	4.83 (1.52)				a-b = −0.69
	Behavioral	4.97 (1.55)				c-b = 3.91 **
Room tidiness–affective scenario	Cognitive	5.14 (1.31)	1.84, 141.31	20.72 **	0.12	c-a = −2.76 **
	Affective	5.65 (1.26)				a-b = 5.54 **
	Behavioral	4.39 (1.61)				c-b = 4.29 **
Room tidiness–behavioral scenario	Cognitive	4.78 (1.47)	1.88, 144.99	28.40 **	0.14	c-a = 3.53 **
	Affective	4.22 (1.52)				a-b = −6.99 **
	Behavioral	5.64 (1.31)				c-b = −4.22 **
Marching drills–cognitive scenario	Cognitive	5.54 (1.10)	2, 154	8.92 **	0.06	c-a = 4.19 **
	Affective	4.83 (1.26)				a-b = −0.48
	Behavioral	4.92 (1.49)				c-b = 3.26 **
Marching drills–affective scenario	Cognitive	4.59 (1.37)	1.87, 144.06	26.35 **	0.13	c-a = −5.48 **
	Affective	5.65 (1.31)				a-b = 6.20 **
	Behavioral	4.47 (1.47)				c-b = 0.77
Marching drills–behavioral scenario	Cognitive	4.87 (1.30)	1.80, 138.34	18.02 **	0.11	c-a = 4.60 **
	Affective	4.05 (1.63)				a-b = −5.01 **
	Behavioral	5.23 (1.28)				c-b = −1.94
Voluntary continuation–cognitive scenario	Cognitive	4.92 (1.31)	2, 154	1.53	0.01	c-a = 1.06
	Affective	4.73 (1.37)				a-b = 0.71
	Behavioral	4.59 (1.52)				c-b = 1.71
Voluntary continuation–affective scenario	Cognitive	4.64 (1.66)	2, 154	23.54 **	0.13	c-a = −5.61 **
	Affective	5.82 (1.23)				a-b = 5.62 **
	Behavioral	4.58 (1.59)				c-b = 0.36
Voluntary continuation–behavioral scenario	Cognitive	4.71 (1.55)	2, 154	9.49 **	0.07	c-a = 0.64
	Affective	4.58 (1.45)				a-b = −4.25 **
	Behavioral	5.44 (1.34)				c-b = −3.13 **

*Note*. ** = *p* < 0.01.

**Table 5:** Overview of the results on comparing the vignettes’ tone in pre-study 2.

Scenario	Vignette	*M* (*SD*)	*DF*	*F*	*η^2^_p_*	*M*_Diff_
Room tidiness	Cognitive	5.56 (1.19)	2, 140	5.99 **	0.04	c-a = −1.84
	Affective	5.86 (1.04)				a-b = 3.31 **
	Behavioral	5.38 (1.09)				c-b = 1.88
Marching drills	Cognitive	5.49 (1.05)	2, 142	17.36 **	0.11	c-a = −3.02 **
	Affective	5.95 (1.01)				a-b = 5.30 **
	Behavioral	5.01 (1.18)				c-b = 3.69 **
Voluntary continuation	Cognitive	5.61 (1.02)	2, 144	12.91 **	0.08	c-a = −4.08 **
	Affective	6.13 (0.88)				a-b = 4.26 **
	Behavioral	5.49 (1.06)				c-b = 0.64

*Note*. ** = *p* < 0.01.

**Table 6:** Means, standard deviations, and correlations of the study variables in the cognitive condition.

Variable	*M*	*SD*	1	2	3	4	5	6	7	8	9	10	11	12	13	14	15	16	17	18	19	20
Proximal cognitive outcomes																						
1. Followers‘ task understanding	4.93	1.15																				
2. Followers‘ perception of task relevance	4.17	1.24	0.68 **																			
3. Followers‘ lack of role ambiguity	5.07	1.10	0.67 **	0.52 **																		
4. Leader clarity	5.60	0.85	0.69 **	0.45 **	0.78 **																	
5. Leader competence	5.40	0.92	0.72 **	0.51 **	0.74 **	0.91 **																
Distal cognitive outcomes																						
6. Followers‘ occupational self-efficacy	4.58	1.38	0.47 **	0.45 **	0.64 **	0.54 **	0.56 **															
7. Followers‘ attitudes toward job tasks	3.60	1.27	0.66 **	0.58 **	0.46 **	0.51 **	0.56 **	0.45 **														
8. Leader endorsement	4.40	1.45	0.54 **	0.32 **	0.62 **	0.66 **	0.68 **	0.57 **	0.54 **													
9. Leader persuasiveness	4.36	1.61	0.69 **	0.47 **	0.61 **	0.64 **	0.69 **	0.56 **	0.65 **	0.74 **												
Proximal affective outcomes																						
10. Followers‘ task motivation	3.36	1.15	0.65 **	0.77 **	0.44 **	0.39 **	0.46 **	0.48 **	0.62 **	0.34 **	0.46 **											
11. Followers‘ sense of belonging	5.50	0.83	0.58 **	0.42 **	0.59 **	0.64 **	0.67 **	0.51 **	0.54 **	0.48 **	0.57 **	0.37 **										
12. Leader warmth	5.21	0.75	0.53 **	0.38 **	0.59 **	0.69 **	0.73 **	0.53 **	0.40 **	0.62 **	0.58 **	0.38 **	0.60 **									
Distal affective outomces																						
13. Followers‘ occupational self-esteem	5.27	1.19	0.61 **	0.50 **	0.69 **	0.71 **	0.72 **	0.69 **	0.55 **	0.57 **	0.57 **	0.51 **	0.62 **	0.52 **								
14. Followers‘ affective commitment	3.79	1.44	0.54 **	0.60 **	0.46 **	0.34 **	0.41 **	0.49 **	0.58 **	0.35 **	0.53 **	0.58 **	0.37 **	0.30 **	0.45 **							
15. Leader liking	4.63	1.54	0.53 **	0.36 **	0.57 **	0.61 **	0.63 **	0.53 **	0.48 **	0.84 **	0.71 **	0.34 **	0.49 **	0.54 **	0.59 **	0.38 **						
16. Leader charisma	4.72	1.31	0.53 **	0.35 **	0.60 **	0.70 **	0.68 **	0.49 **	0.48 **	0.72 **	0.71 **	0.32 **	0.45 **	0.64 **	0.49 **	0.31 **	0.64 **					
Proximal behavioral outcome																						
17. Followers‘ behavioral intention to perform a task	4.62	1.18	0.74 **	0.66 **	0.69 **	0.58 **	0.66 **	0.66 **	0.63 **	0.49 **	0.59 **	0.71 **	0.59 **	0.47 **	0.72 **	0.54 **	0.51 **	0.43 **				
Distal behavioral outcomes																						
18. Followers‘ task performance	5.16	1.19	0.63 **	0.47 **	0.64 **	0.64 **	0.69 **	0.75 **	0.57 **	0.68 **	0.67 **	0.50 **	0.61 **	0.62 **	0.80 **	0.49 **	0.69 **	0.60 **	0.72 **			
19. Followers‘ intention to perform a task	3.71	1.24	0.64 **	0.60 **	0.55 **	0.57 **	0.60 **	0.59 **	0.72 **	0.52 **	0.59 **	0.68 **	0.49 **	0.43 **	0.67 **	0.63 **	0.47 **	0.47 **	0.74 **	0.68 **		
20. Leader following	4.52	1.35	0.66 **	0.43 **	0.66 **	0.64 **	0.67 **	0.58 **	0.57 **	0.83 **	0.76 **	0.45 **	0.53 **	0.55 **	0.68 **	0.47 **	0.79 **	0.67 **	0.63 **	0.77 **	0.62 **	
Control variables																						
21. Attitude toward armed forces	4.32	1.33	0.47 **	0.55 **	0.40 **	0.29 **	0.31 **	0.47 **	0.35 **	0.28 **	0.38 **	0.56 **	0.29 **	0.30 **	0.44 **	0.54 **	0.31 **	0.30 **	0.53 **	0.45 **	0.47 **	0.39 **

*Note.* *M* and *SD* are used to represent mean and standard deviation, respectively. ** indicates *p* < 0.01.

**Table 7:** Means, standard deviations, and correlations of the study variables in the affective condition.

Variable	*M*	*SD*	1	2	3	4	5	6	7	8	9	10	11	12	13	14	15	16	17	18	19	20
Proximal cognitive outcomes																						
1. Followers task understanding	4.69	1.26																				
2. Followers‘ perception of task relevance	4.03	1.30	0.62 **																			
3. Followers‘ lack of role ambiguity	5.04	1.05	0.73 **	0.49 **																		
4. Leader clarity	5.65	0.78	0.52 **	0.19 *	0.46 **																	
5. Leader competence	5.32	0.92	0.57 **	0.17	0.48 **	0.88 **																
Distal cognitive outcomes																						
6. Followers‘ occupational self-efficacy	4.67	1.15	0.51 **	0.29 **	0.49 **	0.33 **	0.41 **															
7. Followers‘ attitudes toward job tasks	3.87	1.37	0.59 **	0.45 **	0.48 **	0.37 **	0.40 **	0.42 **														
8. Leader endorsement	4.42	1.59	0.53 **	0.17	0.41 **	0.53 **	0.60 **	0.46 **	0.59 **													
9. Leader persuasiveness	4.35	1.48	0.61 **	0.32 **	0.46 **	0.47 **	0.54 **	0.45 **	0.64 **	0.79 **												
Proximal affective outcomes																						
10. Followers‘ task motivation	3.37	1.35	0.55 **	0.81 **	0.40 **	0.07	0.10	0.34 **	0.43 **	0.13	0.29 **											
11. Followers‘ sense of belonging	5.53	0.98	0.45 **	0.34 **	0.48 **	0.35 **	0.32 **	0.23 *	0.24 **	0.24 **	0.34 **	0.31 **										
12. Leader warmth	5.59	0.72	0.46 **	0.16	0.38 **	0.76 **	0.73 **	0.25 **	0.23 *	0.34 **	0.31 **	0.15	0.49 **									
Distal affective outcomes																						
13. Followers‘ occupational self-esteem	5.49	0.99	0.59 **	0.37 **	0.40 **	0.42 **	0.50 **	0.54 **	0.49 **	0.39 **	0.46 **	0.40 **	0.45 **	0.45 **								
14. Followers‘ occupational affective commitment	4.19	1.45	0.48 **	0.43 **	0.44 **	0.28 **	0.27 **	0.52 **	0.56 **	0.43 **	0.45 **	0.44 **	0.30 **	0.23 *	0.49 **							
15. Leader liking	4.83	1.72	0.48 **	0.16	0.37 **	0.51 **	0.58 **	0.43 **	0.57 **	0.78 **	0.66 **	0.08	0.35 **	0.44 **	0.44 **	0.40 **						
16. Leader charisma	5.01	1.24	0.55 **	0.19 *	0.46 **	0.56 **	0.55 **	0.41 **	0.47 **	0.71 **	0.62 **	0.16	0.33 **	0.49 **	0.40 **	0.37 **	0.72 **					
Proximal behavioral outcome																						
17. Followers‘ behavioral intention to perform a task	4.71	1.13	0.70 **	0.60 **	0.63 **	0.31 **	0.38 **	0.56 **	0.58 **	0.35 **	0.46 **	0.62 **	0.46 **	0.38 **	0.68 **	0.52 **	0.36 **					
Distal behavioral outcomes																						
18. Followers‘ task performance	5.26	1.11	0.54 **	0.31 **	0.45 **	0.44 **	0.47 **	0.65 **	0.47 **	0.57 **	0.57 **	0.28 **	0.44 **	0.43 **	0.66 **	0.47 **	0.60 **	0.55 **	0.51 **			
19. Followers‘ intention to perform a task	3.96	1.42	0.49 **	0.55 **	0.50 **	0.32 **	0.35 **	0.50 **	0.72 **	0.47 **	0.50 **	0.58 **	0.20 *	0.15	0.48 **	0.53 **	0.38 **	0.34 **	0.57 **	0.45 **		
20. Leader following	4.56	1.45	0.57 **	0.27 **	0.38 **	0.35 **	0.47 **	0.46 **	0.59 **	0.74 **	0.69 **	0.27 **	0.36 **	0.37 **	0.58 **	0.46 **	0.74 **	0.56 **	0.52 **	0.55 **	0.40 **	
Control variable																						
21. Attitude toward armed forces	4.28	1.42	0.42 **	0.62 **	0.37 **	0.03	0.03	0.40 **	0.24 **	0.08	0.11	0.60 **	0.17	0.13	0.43 **	0.48 **	0.02	0.13	0.52 **	0.31 **	0.40 **	0.27 **

*Note.* *M* and *SD* are used to represent mean and standard deviation, respectively. * indicates *p* < 0.05. ** indicates *p* < 0.01.

**Table 8:** Means, standard deviations, and correlations of the study variables in the behavioral condition.

Variable	*M*	*SD*	1	2	3	4	5	6	7	8	9	10	11	12	13	14	15	16	17	18	19	20
Proximal cognitive outcomes																						
1. Followers‘ task understanding	4.73	1.22																				
2. Followers‘ perception of task relevance	4.31	1.20	0.69 **																			
3. Followers‘ lack of role ambiguity	5.28	1.07	0.68 **	0.58 **																		
4. Leader clarity	5.65	0.91	0.50 **	0.37 **	0.63 **																	
5. Leader competence	5.37	0.89	0.55 **	0.41 **	0.62 **	0.91 **																
Distal cognitive outcomes																						
6. Followers‘ occupational self-efficacy	4.64	1.18	0.54 **	0.46 **	0.42 **	0.29 **	0.28 **															
7. Followers‘ attitudes toward job tasks	3.54	1.29	0.47 **	0.46 **	0.39 **	0.24 **	0.37 **	0.37 **														
8. Leader endorsement	4.37	1.55	0.28 **	0.26 **	0.33 **	0.48 **	0.58 **	0.24 **	0.44 **													
9. Leader persuasiveness	4.18	1.40	0.44 **	0.46 **	0.38 **	0.41 **	0.50 **	0.40 **	0.49 **	0.68 **												
Proximal affective outcomes																						
10. Followers‘ task motivation	3.41	1.20	0.59 **	0.69 **	0.39 **	0.21 *	0.28 **	0.51 **	0.57 **	0.22 *	0.41 **											
11. Followers‘ sense of belonging	5.53	0.86	0.50 **	0.39 **	0.62 **	0.57 **	0.61 **	0.26 **	0.34 **	0.42 **	0.37 **	0.35 **										
12. Leader warmth	4.90	0.95	0.39 **	0.27 **	0.35 **	0.66 **	0.72 **	0.28 **	0.33 **	0.48 **	0.42 **	0.20 *	0.47 **									
Distal affective outcomes																						
13. Followers occupational self-esteem	5.45	1.05	0.51 **	0.38 **	0.66 **	0.49 **	0.46 **	0.43 **	0.28 **	0.31 **	0.30 **	0.31 **	0.46 **	0.18								
14. Followers‘ affective commitment	3.93	1.51	0.52 **	0.53 **	0.29 **	0.18 *	0.29 **	0.51 **	0.52 **	0.31 **	0.45 **	0.46 **	0.30 **	0.28 **	0.21 *							
15. Leader liking	4.57	1.64	0.28 **	0.27 **	0.32 **	0.48 **	0.58 **	0.20 *	0.39 **	0.82 **	0.62 **	0.19 *	0.48 **	0.51 **	0.28 **	0.29 **						
16. Leader charisma	4.53	1.29	0.35 **	0.29 **	0.35 **	0.53 **	0.58 **	0.37 **	0.49 **	0.75 **	0.63 **	0.24 **	0.43 **	0.64 **	0.29 **	0.38 **	0.65 **					
Proximal behavioral outcome																						
17. Followers‘ behavioral intention to perform a task	4.77	1.08	0.66 **	0.59 **	0.67 **	0.50 **	0.51 **	0.48 **	0.44 **	0.37 **	0.40 **	0.60 **	0.58 **	0.32 **	0.61 **	0.46 **	0.37 **	0.36 **				
Distal behavioral outcome																						
18. Followers task performance	5.22	1.15	0.52 **	0.47 **	0.63 **	0.47 **	0.52 **	0.49 **	0.44 **	0.49 **	0.51 **	0.46 **	0.52 **	0.36 **	0.70 **	0.32 **	0.49 **	0.46 **	0.58 **			
19. Followers‘ intention to perform a task	3.99	1.23	0.56 **	0.61 **	0.51 **	0.30 **	0.33 **	0.44 **	0.65 **	0.35 **	0.47 **	0.69 **	0.42 **	0.15	0.54 **	0.54 **	0.33 **	0.31 **	0.68 **	0.53 **		
20. Leader following	4.65	1.34	0.44 **	0.37 **	0.45 **	0.58 **	0.59 **	0.41 **	0.46 **	0.80 **	0.62 **	0.31 **	0.46 **	0.56 **	0.44 **	0.35 **	0.73 **	0.73 **	0.51 **	0.58 **	0.45 **	
Control variable																						
21. Attitude toward armed forces	4.22	1.23	0.56 **	0.56 **	0.38 **	0.26 **	0.30 **	0.59 **	0.36 **	0.22 *	0.44 **	0.50 **	0.35 **	0.28 **	0.33 **	0.60 **	0.23 *	0.30 **	0.53 **	0.35 **	0.51 **	0.37 **

*Note.* *M* and *SD* are used to represent mean and standard deviation, respectively. * indicates *p* < 0.05. ** indicates *p* < 0.01.

## Data Availability

The data that support the findings of this study are available at: https://researchbox.org/4357 (accessed on 18 June 2025).

## Supplementary Materials

The following supporting information can be downloaded at: https://www.mdpi.com/article/10.3390/bs15081018/s1, https://researchbox.org/4357, accessed on 18 June 2025. Table S1: Overall outcomes of experts’ assessment of effectiveness of communication techniques. File S1: Written vignettes used in the main study.
