# Supplementary material for: Leader Communication Techniques: Analyzing the Effects on Followers’ Cognitions, Affect, and Behavior
Source: Behav Sci (Basel). 2025 Jul 27;15(8):1018. doi: 10.3390/bs15081018 (PMC12383181; doi:10.3390/bs15081018)
Supplement: Supplementary file 1 [file behavsci-15-01018-s001.zip › Supporting information/Checklist_SelfAssess_Studies_Ethical Safety.pdf]

Checklist

Checklist to Self-Assess Studies Concerning Their Ethical Safety

Each person responsible for a study should fill out this checklist before data acquisition and decide, based on the outcome(s), if an application for approval to the Ethics Committee is necessary.

Titel of the Study: Leader communication techniques: Analyzing the effects on followers' cognitions, affect, and behavior

Principal Investigator (more than one possible): Chantal Utzinger

Responsible Person: Chantal Utzinger

	Yes	No
1. Is there a possibility of disadvantages resulting for participants either through their behavior during the study or through their responses being recorded (e.g. political preferences in non-democratic countries)?	☐	☒
2. Are there any potential disadvantages to not participating in the study?	☐	☒
3. Is participation of persons with limited capacity/incapability of judgment or the participation of minors possible or intended?	☐	☒
4. Will it be necessary for people to partake in the study without consciously knowing so and without previously having given informed consent (e.g. covert observation of people in non-public spaces)?	☐	☒
5. Will participants be deliberately misinformed or falsely informed about the goals and execution of the project (e.g. by manipulated feedback about their performance)?	☐	☒
6. Will participants be asked to disclose personal experiences (e.g. stressful incidents), sensitive information (e.g. sexual behavior, drug use, or attitudes/opinions that could put participants at risk if made public)?	☐	☒
7. In case the physical integrity of participants is affected (e.g. by taking drugs, giving blood samples): Is there a possibility for adverse effects?	☐	☒
8. In case the psychological integrity of participants is affected (e.g. ability to concentrate, induction of negative emotions): Is there a possibility of adverse psychological effects?	☐	☒
9. In case the social integrity of participants is affected (e.g. group experiments): Is it possible for participation to cause adverse effects on the social level (e.g. reputation damage)?	☐	☒

10. Is there an additional financial incentive offered next to the usual compensation for participation in the study? ☐ ☒

11. Does the research funding body, law, or the journal you intend to submit this research to require a registration from an ethics committee? ☐ ☒

12. Has the study already been submitted to an ethics committee for assessment? ☐ ☒

    Name of the committee:
    Date of submission:
    File reference number of approval (if known):

In case of “yes”-answers to any one question 1 through 11, it is mandatory to submit an application for approval of the study to the Ethics Committee (for psychological and related research).

Explanatory Comments on the Questions

Question 1: In cases where responses could put participants at risk (e.g., making controversial political views public in countries where political persecution is possible), it is important to be sure participants are fully protected in terms of data security, confidentiality, and related issues. In cases like these in which anonymity and data security are not fully assured because of the study design and research infrastructure, review may be necessary.

Question 2: In cases where there are dependent relationships between researchers and students (e.g., students who are employed by or graded by members of the research team), it is important to be sure that no negative consequences could arise due to participation or decisions not to participate (e.g., negative employment evaluations or worse grades). In cases like this, there should be a reasonable alternative to study participation and, when practicable, anonymity with respect to this choice in relation to people with power such as employers or instructors.

Question 3: Examples for persons of limited capacity of judgment are children, mentally disabled people or people suffering from dementia or other psychiatric illnesses.

Question 4: This question refers to studies that observe or experimentally influence the behavior of people without their knowledge (for example in field studies or observations of people in non-public spaces).

Question 5: This question refers to studies that deliberately deceive participants (“deception”). That means that participants are purposefully misinformed or falsely informed about integral aspects of the study concerning them, such that they would reasonably perceive themselves as having been lied to once they find out the truth. Among other things, this can include incorrect feedback about their performance, incorrect information about the goals of the study, interactions with a co-worker of the researcher who is falsely introduced as “another study participant”. This question does not refer to the

fact that usually study participants aren't fully informed about the scientific background or the hypotheses of a study.

Question 6: This question refers to data acquisition that is sensitive due to one of two reasons. On the one hand, it's about information that needs to be treated highly confidentially because its distribution could lead to disadvantages for the person. On the other hand, it's about disclosed information that can be connected with strong emotions for the person (e.g. traumatic experiences) in a way that could cause the assessment to be an unacceptable emotional strain.

Question 7: This question refers to physical interventions, for example taking drugs (including alcohol) as well as invasive measures like taking blood samples, injection of contrasting agents. Unproblematic physical interventions are things like drinking non-alcoholic beverages, moderate exercise, measuring blood pressure.

Question 8: Just as in question 7, it's about distinguishing between negative and neutral consequences of the intervention. For example mood induction with happy or sad music is harmless because this type of music is common in everyday life and no adverse effects are to be expected. What would be disquieting or disturbing would be showing images of war or mutilation – it's true that those types of pictures can also be found in everyday life but usually one is not forced to look at them and they can be cause for very strong emotional reactions.

Question 9: Not every group experiment is ethically questionable but under certain conditions group experiments can possess a risk of placing people in uncomfortable situations, for example if an experiment creates a competitive situation in which individual participants will clearly lose, if aggression is induced, or if people consider the situation to be embarrassing. Just like in the two previous questions, here one must also draw a line between a small discomfort which can be considered comparable to an everyday occurrence and thus acceptable (e.g. nervousness some people always feel when talking in front of a group) and a discomfort that oversteps the boundary of what can be considered acceptable (e.g. being yelled at).

Question 10: Here the distinction is to be made between the standard compensation for a respective field of research for study participation and a financial incentive used specifically in the study to reach a certain goal of the study (e.g. particularly high performance motivation).

Question 11: If a research funding body, the journals to which the researcher tends to submit the results of the study, or the law requires an ethical review/and or registration, we can provide a review even for studies that would otherwise be exempt.

Question 12: This question refers to ethics committees other than the one of the Faculty of Philosophy, e.g. out-of-canton ethics committees involved in multi-centered studies.

In general: If in doubt, consult a member of the Ethics Committee of the Faculty of Philosophy.
