# Supplementary material for: Leader Communication Techniques: Analyzing the Effects on Followers’ Cognitions, Affect, and Behavior
Source: Behav Sci (Basel). 2025 Jul 27;15(8):1018. doi: 10.3390/bs15081018 (PMC12383181; doi:10.3390/bs15081018)
Supplement: Supplementary file 1 [file behavsci-15-01018-s001.zip › Supporting information/File_S1.pdf]

Supporting information

Written vignettes used in the main study

Vignettes are originally in German and double translated with DeepL.

Scenario room tidiness

Cognitive vignette (high cognitive, low affective, and low behavioral)

Imagine your platoon leader, Lieutenant Schmid, calls you and your comrades out of your room. You stand together in front of him, and he says that you have not followed the room regulations. He then says the following:

“I would like to explain to you why the room regulations are so important. (explaining) I want you to understand that the room regulations have a purpose. (explaining) In the Swiss Armed Forces, room regulations are relevant for the following two reasons: Firstly, operational readiness and secondly, hygiene.

First of all, it's important that your room is always tidy because this means you can always find your equipment and are quickly ready for exercises in the event of an alarm. (explaining)

The second reason is hygiene: our everyday life primarily takes place outside. We sweat, get dirty and sometimes wet. But to prevent viruses and bacteria from spreading in the rooms, it is important that the rooms are always clean and tidy. (explaining)

I don't want to burden you unnecessarily with the room regulations, but that you understand why they are so important. (explaining) Your focus on the room regulations will make a difference and contribute to the readiness of the Armed Forces and to the health of all of you. (explaining) I think it is precisely because of this sense of solidarity that it is important and right to keep to the room regulations! (expressing moral conviction)

What exactly are the rules of the room regulations? (information request) I would like to briefly explain to you once again what the room regulations mean. Room regulations mean

that your things are clean and stowed away, that your beds are made, and the floor is mopped. (explaining)

To ensure that you can adhere to the room rules in the end, it is important that you divide up the tasks among yourselves and define exactly who is responsible for which part of the room regulations. (explaining) This division of responsibility will help you with your time management, among other things, because you will not have to do a lot of work once, but rather a little more than once. (explaining) Is this a comprehensible procedure for you? (confirmation request)

But before you start, can you summarize the most important arguments why it is important that we comply with the room regulations? (summary question)"

Total (in the original German vignette): 282 words, 17 sentences, 13 leader communication techniques (5 different types of leader communication techniques)

Affective vignette (low cognitive, high affective, and low behavioral)

Imagine your platoon leader, Lieutenant Schmid, calls you and your comrades out of your room. You stand together in front of him, and he says that you have not followed the room regulations. He then says the following:

"I would like to talk to you about the room regulations and encourage you to keep to them in future. (encouragement) For you, an untidy room is probably not that bad and you probably even find tidying up a bit of a chore. It was the same for me at the beginning of recruit school. (agreeing)

I can understand you and I also agree that this is not the most important part of your everyday life. (agreeing) From my point of view, it even makes a lot of sense for you to prioritize! (praising others) But the room regulations are an important part of our agenda, and

we have to stick to them. It is not my aim to annoy you with the room regulations, quite the opposite! I would like to encourage you to keep at it on this point too, because what you have shown so far in basic military training is very good. (praising others) In view of your achievements so far, I know how committed you are and how much you can achieve even under difficult conditions. (expressing confidence that goals can be achieved) Keep this commitment to tidying up your room! (encouragement)

Order is a sign of appreciation and respect for your comrades in the room. I notice in everyday life and in military exercises that you respect and appreciate each other. (praising others) You can express this respect and appreciation to your comrades even more strongly by maintaining order and cleanliness in your room. (encouragement) I know that you will manage this in this area too. (expressing confidence that goals can be achieved)

I agree with you that this sounds a bit strange at first (agreeing): The room regulations should lead to more appreciation and respect. But believe me, by adhering to the room regulations, we ensure that we show consideration for each other.

But before you start: If you set yourself the goal of adhering to the room regulations, I'm sure you'll achieve it! (expressing confidence, that goals can be achieved; during whole vignette eye contact)"

Total (in the original German vignette): 282 words, 17 sentences, 13 leader communication techniques (5 different types of leader communication techniques)

Behavioral vignette (low cognitive, low affective, and high behavioral)

Imagine your platoon leader, Lieutenant Schmid, calls you and your comrades out of your room. You stand together in front of him, and he says that you have not followed the room regulations. He then says the following:

“Come to me, everyone, we'll talk about the room regulations together. (command) We'll talk about what you have to do in future to comply with the room regulations. Because our goal is for you to pass every room inspection by the end of recruit school. (goal setting)

So, everyone listen to me carefully! (command) I want each of you to know what you can do personally to do your part to keep your room tidy. (goal setting) Tidy up your things regularly, put them away in the appropriate places and clean the room. (giving precise instruction) I suggest that you all work together towards this goal (making a proposition) and therefore I make the following suggestion: talk to your room manager and he will then divide the various tasks equally between you. (making a proposition) For example, one person makes sure that the floor is always clean and mopped. Another person checks that the snacks are stowed away and not lying around openly. Every morning and every evening, as the person responsible for the office, you check the entire room and point out any untidiness. (giving precise instruction) All other roommates listen to these requests and then implement them directly. (giving precise instruction)

Let's get started: you now have 10 minutes to get your room in order! (command) That means, first of all, you divide up the tasks among yourselves. (giving precise instructions) If you wish, you can also change the tasks on a weekly basis. (making a proposition) Then go to your room, where you organize and hang up your clothes. Then put your personal belongings away and finally make your bed and mop the floor. (giving precise instructions)

But before you start, please repeat for me: What are your next steps - i.e. what are you going to do now - to comply with the room regulations? (control question)"

Total (in the original German vignette): 283 words, 17 sentences, 14 leader communication techniques (5 different types of leader communication techniques)

Scenario marching drills

Cognitive vignette (high cognitive, low affective and low behavioral)

Imagine you are standing in front of your platoon leader, Lieutenant Schmid. You have already seen on the picasso that marching drills are coming up. Your platoon leader looks at you and your comrades and says the following:

"Before we move on to the training ground, let's take a look at the marching drills. To start with, I would like to explain to you in detail that marching drills are not about simply chasing you around the range. Rather, it's about practicing formations in a structured way. (explaining)

I want you to understand why we are doing this in the first place: The marching drills are the presentation form of the platoon. In other words, it represents discipline and platoon cohesion. (explaining) These two points are essential in the Armed Forces, and I think it's important to know them. (expressing moral conviction)

Let me explain what the marching drills will teach you: First, you learn how to work together in a platoon. This means you learn how important it is to think with and for your colleagues in a team. This means that you are constantly learning and improving by working together. (explaining) Secondly, the platoon school promotes discipline. It's not just about adhering to rules and regulations, but also about your own concentration and focus on the goal. (explaining) And this is a skill worth mastering (expressing moral conviction), because

it will not only help you in the military, but also for your private and professional goals. (explaining) With each additional training block, you will learn more and improve significantly in these skills.

What does that mean for you in summary? (summary question) If everyone sticks to their steps and positions on the train, it works in the end. An important prerequisite for this is that everyone is attentive and understands why we are doing this. (explaining) It's about your understanding of the meaning and purpose of the individual commands, which is why we're going through them now and I'm going to explain them to you. (explaining) Does this procedure make sense to you? (confirmation request)

Before we start with the first exercises of the marching drills, I would like you to answer two questions. How exactly did we set up last time? (information request) And in summary, what are the most important arguments in favor of practicing the marching drills? (summary question)"

Total (in the original German vignette): 318 words, 21 sentences, 13 leader communication techniques (5 different types of leader communication techniques)

Affective vignette (low cognitive, high affective, and low behavioral)

Imagine you are standing in front of your platoon leader, Lieutenant Schmid. You have already seen on the picasso that marching drills are coming up. Your platoon leader looks at you and your comrades and says the following:

"Before we move on to the training ground, let's focus on marching drills. You have already practiced marching and reaction exercises well in the previous training sessions. (praising others) I already know that we can take another step towards successful marching drills today. (expressing confidence, that goal can be achieved)

You probably don't have much desire to run around the field in uniform and with a weapon. My former recruit self would immediately agree with you (agreeing), because back then my motivation for marching drills was also limited. I had no desire to run around pointlessly. That's not what gets us anywhere either, I still see it that way today! (Agreeing) We prefer to concentrate on the important aspect of the marching drills: strengthening the team spirit in our platoon. You're already a well-rehearsed team (praising others), but we can improve on that! (encouragement)

Each and every one of you is an important part of the various formations and only together can we make the formations look good. This works if you have confidence. Confidence in yourself, your comrades - in all of us! This trust is the basis for successful cooperation during marching drills and in everyday military life. This is exactly what we want to achieve here with our platoon, and I am convinced that we can do it. (expressing confidence, that goal can be achieved)

So far you have been committed to the exercises and have performed well (praising others), keep up the good work! (encouragement) We don't have to do everything perfectly straight away; I agree with you. (agreeing) But if we keep at it, we will succeed in implementing the formations and we will have fun! It may seem tedious and boring now, but as soon as you get the formations right, you'll have a good feeling. So, keep at it and don't give up! (encouragement)

Before we start with the first exercises of the marching drills, I would like to tell you that you will continue to improve with training, and I am convinced that you will find it easier each time! So, give it your all! (encouragement; constant eye contact)"

Total (in the original German vignette): 318 words, 21 sentences, 13 leader communication techniques (5 different types of leader communication techniques)

Behavioral vignette (low cognitive, low affective, and high behavioral)

Imagine you are standing in front of your platoon leader, Lieutenant Schmid. You have already seen on the picasso that marching drills are coming up. Your platoon leader looks at you and your comrades and says the following:

“Before we move on to the training ground, let's focus on the marching drills. So, everyone, come here and listen carefully! (command) I would like to tell you what your two main tasks are for today's marching drills and what steps you will take to tackle them: First of all, you always follow my commands. As soon as I give you a command, for example, “in column of two - march”, you carry out this command precisely. (giving precise instruction) Another task is marching. On my command “forward - march!”, you all start at the same time with your left foot, then follow with your right foot and then march neatly in step. (giving precise instruction)

The main goal of our platoon is to achieve the best possible rating in the next inspection. (goal setting) An important part of this inspection will be the marching drills. I want you to listen to my commands, keep to all your steps and positions and ensure that all movements are synchronized at the end. (goal setting) We will work specifically on this today and in all upcoming training sessions. I suggest that we start slowly and increase the pace as soon as you can perform the exercises correctly. (making a proposition)

I want us to improve with each training block and practice increasingly difficult formations. (goal setting) Therefore, my suggestion is that today's training block runs as follows: We practice all three types of exercises, one after the other. (making a proposition) First we start with the reaction exercises. On my command, you all turn and run in the same direction. (giving precise instruction) As soon as you have mastered the reaction exercises, we will continue with the collection exercises. You will also stand correctly on my command

for these exercises. (giving precise instruction) Finally, we will practice marching in step. My aim is that at the end of this block you will always know exactly what you have to do for the three types of exercises in the marching drills. (goal setting)

Before we start with the first exercises of the marching drills, I would like you to repeat them once again: What do you actually do during the reaction exercises? (control question) Then do exactly that now, get started! (command)”

Total (in the original German vignette): 317 words, 21 sentences, 13 leader communication techniques (5 different types of leader communication techniques)

Scenario: voluntary continuation after basic military training

Cognitive vignette (high cognitive, low affective, and low behavioral)

Imagine that you and your comrades have to wait for the Duro to take you to your platoon workstation. While you are waiting, your platoon leader, Lieutenant Schmid, stands up and asks to speak to you. He starts the conversation as follows:

“I would like to use the waiting time to explain to you specifically why you should continue after basic military training and why you can benefit from it. (explaining) You've probably already asked yourself what training as a group leader would bring you? (confirmation request) Let me explain it to you in detail: On the one hand, you acquire specific knowledge at non-commissioned officer (NCO) school, on the other hand you learn to take on leadership responsibility and in the process, you are trained to become a good group leader. With this acquired knowledge, you will then have all the prerequisites to be able to lead your recruits successfully. (explaining) You do not have to have a professional military career as your goal, because even as a conscription officer you will be given the opportunity to receive unique leadership training. (explaining) However, continuing your

training after the basic military training, or training in the NCO school, also creates an ideal starting position for your civilian career. (explaining) Let me explain it to you: Imagine, for example, that you are in charge of a complex project, or you have to make a professional decision. At NCO school, you learn the military leadership skills that help you to make targeted decisions and find feasible solutions to problems, even under difficult conditions. (explaining) There is probably no other place where you have the opportunity for such training. Do you agree? (confirmation request)

What are your current thoughts about continuing after basic military training? (information request)

It is not only for your personal development, but also for the security of society that it is right and important to serve in the military. (expressing moral conviction) Because in the end, not only will you be able to continue your education at NCO school, but you will also be helping the Swiss Armed Forces to fulfill their three main tasks: National defense, subsidiary support for the civilian authorities and peace support. (explaining) And for all these reasons, I think it is right to decide to continue. (expressing moral conviction)

To ensure that you consider all key aspects when making your decision, let's think this through further at a later date. (explaining) I would also like to use this conversation to clarify any questions you may have about how you will benefit from continuing. (explaining) Is that an understandable approach for you? (confirmation request)

Before we get into the Duro, can you summarize for me what arguments speak for you personally to continue? (summary question)"

Total (in the original German vignette): 355 words, 18 sentences, 15 leader communication techniques (5 different types of leader communication techniques)

Affective vignette (low cognitive, high affective, and low behavioral)

Imagine that you and your comrades have to wait for the Duro to take you to your platoon workstation. While you are waiting, your platoon leader, Lieutenant Schmid, stands up and asks to speak to you. He starts the conversation as follows:

“I would like to use the waiting time to show you how good your work is, how important you are to the army and how much I would like to have you in the non-commissioned officer (NCO) school. (praising) You probably think that you can't benefit at all from NCO school. I probably would have agreed with you as a recruit back then (agreeing), but today I can tell you with conviction that this is not true. On the contrary, I even encourage you to go to NCO school. (encouragement) At NCO school, you will not only experience numerous valuable situations that will boost your self-confidence. If you set yourself the goal of successfully completing NCO school and further developing your leadership skills, then I have no doubt that you will succeed. (confidence, that goals can be achieved)

I agree with you that you don't have to pursue a career as a professional military officer (agreeing), because even as a conscription officer you benefit from unique leadership training and can use your strengths effectively. (praising) I know your many strengths and your ability to take care of your comrades. I am sure that you would successfully complete NCO school and then, as a group leader, provide valuable military service for the recruits in your group. (Confidence, that goals can be achieved) Stay as committed as ever. (encouragement) I know and am convinced that you will do very well! (Confidence, that goals can be achieved)

You may think that your work won't make any difference at all, but it certainly will! I realize that it may sound a bit exaggerated (agreeing), but basic military training is a very formative time for recruits. But I also know that you will be able to help shape this formative

time in a positive way and give the recruits in your group a sense of achievement. (Confidence, that goals can be achieved)

You are probably experiencing different feelings about doing nCO school voluntarily. I understand that, because I wasn't completely sure at the time whether I should really do NCO school. (agreeing) But then the challenge really appealed to me and I'm sure that you would master it too. (confidence, that goals can be achieved) Let's tackle this together and discuss it in more detail at a later date and we can clarify any questions you still have.

Before we get into the Duro now, I would like to say to you: keep doing exactly what you are doing! (encouragement; constant eye contact) ”

Total (in the original German vignette): 353 words, 19 sentences, 15 leader communication techniques (5 different types of leader communication techniques)

Behavioral vignette (low cognitive, low affective, and high behavioral)

Imagine that you and your comrades have to wait for the Duro to take you to your platoon workstation. While you are waiting, your platoon leader, Lieutenant Schmid, stands up and asks to speak to you. He starts the conversation as follows:

“Come and see me! (command) I would like to use the waiting time to talk to you about moving on. To be more precise, I would like to suggest that you complete non-commissioned officer (NCO) school (making a proposition), because it is clear to me that you should continue. You don't have to aim for a professional military career; as a conscription officer, you will also have the opportunity to receive one-off leadership training.

My goal, however, is that you will consciously put the option to continue into practice. (goal setting) To find out whether you want to continue, carry out the following steps: (giving precise instruction) Gather information about selection, NCO school and

earning practical service. (giving precise instruction) I suggest that you talk to the group leaders about their experiences. (making a proposition) Ask them what their specific tasks as a squad leader are and what exactly the training content of the NCO school is (giving precise instruction). Also talk to your family and friends. (giving precise instruction) And if you decide to continue, which I very much support, then work from now on to ensure that you are well prepared (goal setting) and that you successfully complete NCO school (goal setting).

If you want to keep going, then do this in the coming weeks of basic military training: Show everyone that you are ready to perform. For example, you can push yourself to your limits with every exercise in the sports units and do an extra repetition, even if you have no strength left. (giving precise instruction) You can also use team tasks to show that you can both cooperate with and convince your teammates during the exercises. (giving precise instruction) The same applies to taking responsibility. For example, lead the parking service when the cadre is not on site and pay attention to how this makes military life easier for your comrades. (giving precise instruction)

I suggest that we discuss what it means for you to pursue a cadre career at the next meeting. (making a proposition) After you have spoken to those around you, ask me any questions you still have in our meeting, and I will try to answer them. (giving precise instruction)

Before we get into the Duro now, please repeat again for control: What is your task until the next meeting and what are your next steps to come to a decision? (control question)"

Total (in the original German vignette): 356 words, 18 sentences, 16 leader communication techniques (5 different types of leader communication techniques
