# Supplementary material for: Leader Communication Techniques: Analyzing the Effects on Followers’ Cognitions, Affect, and Behavior
Source: Behav Sci (Basel). 2025 Jul 27;15(8):1018. doi: 10.3390/bs15081018 (PMC12383181; doi:10.3390/bs15081018)
Supplement: Supplementary file 1 [file behavsci-15-01018-s001.zip › Supporting information/Table_S1.pdf]

Supporting information

Table S1

Overall outcomes of experts' assessment of effectiveness of communication techniques.

Communication technique	Effectiveness		
	Cognitive *M* (*SD*)	Affective *M* (*SD*)	Behavioral *M* (*SD*)
Verbal techniques			
Questions			
Control question	**4.00 (1.00)**	2.71 (0.95)	***4.29 (0.76)***
Suggestion question	**4.00 (1.15)**	**3.57 (1.13)**	3.43 (1.27)
Confirmation request	***4.00 (0.82)***	**3.57 (1.27)**	**3.71 (1.11)**
Asking open questions	**3.86 (1.07)**	**3.71 (0.95)**	2.43 (1.40)
Information request	***4.00 (0.58)***	2.71 (1.60)	**4.14 (0.90)**
Alternative question	2.71 (1.11)	2.29 (1.89)	**3.71 (1.50)**
Summary question	***4.71 (0.49)***	3.00 (1.29)	2.29 (0.76)
Rhetorical question	2.29 (1.38)	3.43 (1.72)	2.57 (1.51)
Statements			
Agreeing	2.86 (1.46)	***4.71 (0.49)***	2.71 (1.25)
Explaining	***4.43 (0.79)***	3.29 (1.25)	**3.71 (1.50)**
Expressing moral conviction	***4.14 (1.46)***	**4.29 (1.11)**	**3.71 (1.25)**
Giving precise instructions	**4.00 (0.82)**	2.50 (1.22)[1]	***5.00 (0.00)***
Praising others	3.29 (0.49)	***4.86 (0.38)***	3.29 (0.49)
Short phrases	3.20 (1.10)[1]	3.00 (1.58)[1]	2.00 (1.00)[1]
Three-part lists	**3.50 (1.38)**[1]	3.17 (1.17)[1]	2.83 (1.33)[1]
Comparisons	3.29 (1.50)	3.43 (1.27)	3.00 (1.29)
Contrasts	3.14 (1.21)	3.43 (0.98)	**3.57 (1.27)**
Confidence goal achieving	2.86 (1.57)	***4.71 (0.49)***	**4.29 (0.49)**
Expressing sentiment of collective	2.00 (1.15)	3.43 (1.51)	2.57 (1.27)
Making a proposition	3.29 (1.11)	2.71 (0.95)	***4.71 (0.76)***
Personal pronouns	3.29 (1.38)	**4.00 (1.15)**	**3.86 (1.07)**
Setting goals	3.29 (1.11)	3.00 (0.82)	***4.86 (0.38)***
Speaking in pictures	3.00 (1.00)	**4.14 (0.90)**	3.14 (1.35)
Speaking with stories	3.14 (1.07)	**4.29 (0.76)**	2.57 (0.79)
Imperative sentences			
Command	2.71 (0.49)	2.71 (0.76)	***4.57 (0.79)***
Encouragement	2.71 (0.95)	***4.43 (0.53)***	**4.14 (0.90)**
Paraverbal techniques			
Voice			
Accent chains	2.86 (1.07)	3.43 (1.72)	2.71 (0.95)
Fluency	3.14 (0.90)	3.14 (0.38)	2.71 (0.76)
Clear pronunciation	3.43 (0.98)	3.14 (0.38)	2.71 (0.49)
Positive intensification	2.33 (0.82)[1]	3.33 (1.37)[1]	2.33 (0.82)[1]
Variation in loudness	2.43 (0.98)	**3.71 (1.60)**	2.17 (0.98)[1]
Clearing throat	2.67 (1.37)[1]	2.83 (2.04)[1]	3.17 (1.33)[1]
Intended pauses	3.43 (1.13)	**3.57 (1.51)**	2.57 (1.40)
Reinforcement	1.83 (0.98)[1]	3.17 (1.33)[1]	1.83 (0.75)[1]
Higher speaking rate	2.57 (1.51)	2.71 (0.95)	2.43 (0.53)
Word repetition	3.00 (0.63)[1]	**3.50 (0.55)**[1]	2.14 (1.07)
Non-verbal techniques			
Mimics and Gestures			
Body gestures	3.14 (1.35)	**3.86 (1.07)**	2.86 (1.07)
Hand & arm gestures	2.86 (1.35)	3.29 (1.50)	2.50 (1.05)[1]
Indicating directions	2.71 (0.95)	3.29 (1.11)	2.86 (1.21)
Nodding	3.00 (1.10)[1]	3.33 (1.21)[1]	3.17 (1.33)[1]
Eye contact	3.00 (1.29)	***4.86 (0.38)***	**3.86 (1.07)**
Head position	2.83 (1.33)[1]	**4.00 (0.89)**[1]	3.00 (1.41)[1]

Intensity of facial expression	2.43 (0.79)	**4.43 (0.79)**	3.14 (0.69)
Smiling	2.29 (0.95)	**3.86 (1.35)**	2.57 (0.79)

Note. Bold numbers are above the cut-off 3.5

Italic numbers are among the five most effective techniques

[1] Some experts answered "I have no idea" on the effectiveness item
